# Proteomics and Metabolomics Analyses to Elucidate the Desulfurization Pathway of *Chelatococcus* sp.

**DOI:** 10.1371/journal.pone.0153547

**Published:** 2016-04-21

**Authors:** Naba K. Bordoloi, Pabitra Bhagowati, Mihir K. Chaudhuri, Ashis K. Mukherjee

**Affiliations:** ONGC-Center for Petroleum Biotechnology, Department of Molecular Biology and Biotechnology, Tezpur University, Tezpur, 784028, Assam, India; East China Normal University, CHINA

## Abstract

Desulfurization of dibenzothiophene (DBT) and alkylated DBT derivatives present in transport fuel through specific cleavage of carbon-sulfur (C-S) bonds by a newly isolated bacterium *Chelatococcus* sp. is reported for the first time. Gas chromatography-mass spectrometry (GC-MS) analysis of the products of DBT degradation by *Chelatococcus* sp. showed the transient formation of 2-hydroxybiphenyl (2-HBP) which was subsequently converted to 2-methoxybiphenyl (2-MBP) by methylation at the hydroxyl group of 2-HBP. The relative ratio of 2-HBP and 2-MBP formed after 96 h of bacterial growth was determined at 4:1 suggesting partial conversion of 2-HBP or rapid degradation of 2-MBP. Nevertheless, the enzyme involved in this conversion process remains to be identified. This production of 2-MBP rather than 2-HBP from DBT desulfurization has a significant metabolic advantage for enhancing the growth and sulfur utilization from DBT by *Chelatococcus* sp. and it also reduces the environmental pollution by 2-HBP. Furthermore, desulfurization of DBT derivatives such as 4-M-DBT and 4, 6-DM-DBT by *Chelatococcus* sp. resulted in formation of 2-hydroxy-3-methyl-biphenyl and 2-hydroxy –3, 3^/^- dimethyl-biphenyl, respectively as end product. The GC and X-ray fluorescence studies revealed that *Chelatococcus* sp. after 24 h of treatment at 37°C reduced the total sulfur content of diesel fuel by 12% by per gram resting cells, without compromising the quality of fuel. The LC-MS/MS analysis of tryptic digested intracellular proteins of *Chelatococcus* sp. when grown in DBT demonstrated the biosynthesis of 4S pathway desulfurizing enzymes viz. monoxygenases (DszC, DszA), desulfinase (DszB), and an NADH-dependent flavin reductase (DszD). Besides, several other intracellular proteins of *Chelatococcus* sp. having diverse biological functions were also identified by LC-MS/MS analysis. Many of these enzymes are directly involved with desulfurization process whereas the other enzymes/proteins support growth of bacteria at an expense of DBT. These combined results suggest that *Chelatococcus* sp. prefers sulfur-specific extended 4S pathway for deep-desulphurization which may have an advantage for its intended future application as a promising biodesulfurizing agent.

## Introduction

Sulfur is one of the most abundant elements present in crude petroleum- oil. Release of harmful sulfur oxides (SO_2_) in the environment through the combustion of fossil fuels contributes to the air pollution that imposes a serious eco-hazard problem worldwide [1, 2]. Moreover, sulfate particulate matter present in transportation fuel also decreases the life of motor engine through corrosion. Therefore, as per the regulation, the most widely used transport fuel, for example diesel, should contain ultra-low levels (<15 ppm) of sulfur. In order to minimize the sulfur content as well as environmental pollution, oil refineries use a conventional chemical process known as hydrodesulfurization (HDS) to convert the sulfur of the fossil fuel to a less polluting compound hydrogen sulfide [3]. Nevertheless, this technique requires high pressure (1–20 MPa) and high temperature (290–450°C) resulting in big capital investment, high operating costs, and in some instances also leads to deterioration of fuel quality [4, 5]. Although the aliphatic sulfur compounds can efficiently be removed through HDS treatment; however, the most abundant heterocyclic thiophenic compounds such as dibenzothiophene (DBT) and alkyl substitutions at 4 and 6 positions of DBT (4,6-dimethyldibenzothiophene) present in transportation fuels are refractory to HDS treatment and therefore hard to remove from the petroleum oil [1,2,4]. Consequently, an alternative green technology to mitigate the problems associated with the HDS process would be a welcome measure to reduce the environmental pollution emerging from fossil fuel combustion.

The biodesulfurization (BDS), an alternate choice to HDS, offers the potential for a more selective and cost-effective method for reducing the sulfur content from the refractory organic compounds of fuels under mild processing conditions [6]. Moreover, emission of CO_2_ is significantly lowered in BDS process resulting in the reduction in the emission of greenhouse gases. Microbes have long been known to utilize the components of crude petroleum-oil to sustain their growth and survival in that environment [7, 8]. Microbial desulfurization or biodesulfurization (BDS) is considered as an eco-friendly, cost-effective green technology to overcome the technical as well as high operation cost associated with the conventional HDS treatment [4, 9]. DBT has been set as a model substrate compound for the isolation of promising bacterial strain(s) capable of transforming organosulfur compounds (deep-desulfurization) found in a variety of fossil fuels [4, 9]. The desulfurizing bacteria have evolved diverse biochemical pathways in order to utilize sulfur from the DBT and its derivatives to sustain their growth as well as to obtain energy [4]. The two major DBT utilization pathways operate in bacterial system are- (a) ring destructive (degradation) or “Kodama pathway”, in which initial dioxygenation is carried out at the peripheral aromatic ring of DBT followed by cleavage of the ring leading to the formation of 3-hydroxy–2-formylbenzothiophene as the end product, and (b) sulfur-specific (desulfurization) or “4S pathways”, in which DBT is desulfurized and finally converted to 2- hydroxybiphenyl (2-HBP). Few of the researchers have demonstrated further that in the extended 4S pathway operating in some bacterial system the 2- HBP formed from DBT is partially converted to 2-methoxybiphenyl (2-MBP) albeit the exact mechanism and enzyme(s) involve in this conversion remain unknown [9–12]. Advantage of 4S pathway of desulfurization is in this process the carbon skeleton of DBT remains intact without compromising the calorific value of the fuel [1, 9, 13]. Therefore, bacteria following 4S pathway for BDS are of great industrial demand for large scale deep-desulfurization of fossil fuel [9, 10].

The DBT desulfurizing potential of different bacteria, for example, *Rhodococcus erythropolis* XP, *R*. *erythropolis* IGTS8, *R*. sp. ECRD-1, *Gordonia alkanivorans* RIPI90A, *Mycobacterium* sp. X7B, and *Achromobactor* sp. has been well established [1, 2, 9, 11, 14]. Most of the above bacteria follow 4S pathway for BDS that leads to the formation of 2-HBP and/or 2-MBP and sulfate as the end products. However, accumulation of 2-HBP inhibits the enzymes associated with BDS that results in limited cell growth and subsequently exerts a negative effect on BDS process [15, 16]. Besides, several other key factors, for example, cellular level of reducing powers (NADH and FMNH_2_), O_2_ level of cytoplasm, transmemebrane trafficking of substrate (DBT) and/or its toxic end products may take a decisive role in determining the BDS efficiency of a microorganism [17]. Therefore, the major challenge faced by the oil refineries for the development of a suitable commercial BDS process is the isolation of a strain with higher BDS activity (deep-desulfurization) without compromising the quality of crude oil. In this communication we are for the first time reporting the biocatalytic DBT desulfurization pathway of a new bacterium *Chelatococcus* sp. isolated from a petroleum-oil contaminated soil. This bacterium can grow in a wide range of thiophenic compounds, and it produces 2-MBP instead of 2-HBP as the end product which is much less toxic to bacteria and also reduces environmental pollution. Our results substantiate the potential application of the bacterium under study for the biocatalytic desulfurization of diesel fuel under mesophilic growth conditions which has the added economical advantage to reduce the energy consumption during BDS in addition to reduce the environmental pollution.

## Materials and Methods

### Chemicals

DBT, 4M-DBT, 4, 6-DM-DBT and 2-HBP were purchased from Sigma-Aldrich. All other analytical grade chemicals were purchased from Merck (Germeny). HDS treated diesel oil was collected from Indian Oil Corporation Ltd. fuel station (Tezpur). Crude petroleum oil was collected from Borhola oil fields (26.44° N, 94.15°E) of Oil and Natural Gas Corporation Limited, Assam, India. The sample was collected under Memorandum of Understanding between Tezpur University and ONGCL to pursue scientific research in the area of Petroleum Biotechnology.

### Isolation of bacterial strain and pure culture

The isolation of desulfurizing bacterial strains from crude petroleum-oil contaminated soil samples, collected from oil fields of NE India, was based on their ability to utilize DBT as the sole source of sulfur. Pure cultures of bacteria were screened for their ability to grow on basal salts medium (BSM) supplemented with 0.5 mM DBT dissolved in *N*, *N* -dimethylformamide (DMF) as the sole source of sulfur. The composition of BSM was (L^-1^): 4.0 g glycerol, 2.44 g KH_2_PO_4_, 14.04 g Na_2_HPO_4_, 2.0 g NH_4_Cl, 2.0 mL of 10% (w/v) MgCl_2_, 100 μL of 1% (w/v) FeCl_3_, 100 μL of 1% (w/v) CaCl_2_, 200 μL of vitamins mixture and 5.0 mL of metal solution [9,14]. Single bacterial colony isolation from turbid cultures was performed by plating appropriately diluted culture samples onto Luria broth (LB) medium composed of 10.0 g Bactotryptone (Difco), 5.0 g of yeast extract (Difco), 10.0 g of NaCl in 1000 mL of distilled water (pH adjusted to 7.0) and supplemented with 15.0 g of Bacto Agar (Difco). The bacterial strains were sub-cultured on LB agar plates before use as inoculums and stored in glycerol at -80°C.

Replicated batch cultures were grown in 250 mL Erlenmeyer flasks containing 100 mL of BSM supplemented with DBT (0.5 mM) as the sulfur source. Kinetics of bacterial growth by utilizing the above mentioned compounds was assessed by measuring the bacterial cell population at 600 nm, protein concentration of culture supernatant and bacterial dry biomass [8] with respect to time. All flasks were kept in a rotary shaker incubator at 37°C, pH 7.0 at 200 rpm. Non-inoculated flasks and flasks without sulfur source were served as controls.

### Identification of bacterial strain

Bacterial identification was done by using the standard microbial identification procedure viz. (a) studying the morphological characteristics and phenotypic properties of bacteria, and (b) 16S rRNA gene sequencing followed by phylogenetic analysis.

#### 16S rRNA gene sequencing and phylogenetic analysis

The bacterial genomic DNA was isolated according to the procedure of Ausubel et al. [18]. Briefly, the conserved region of 16S rRNA gene was amplified by polymerase chain reaction (PCR) using the universal primers that were designed to amplify a 1,538-bp segment of the 16S rDNA. The forward primer was: 5’- GAG TTT GAT CCT GGC TCA G -3’, and the reverse primer was: 5’- CGG CTA CCT TGT TAC GAC TT -3’. PCR was carried out as described by Rahman et al. [19] using PCR system thermal cycler (GeneAmp® PCR system 9700, Applied Biosystems). The amplified DNA fragment (1.5 kb) was separated on 1% (w/v) agarose gel, stained with ethidium bromide, eluted from the gel using a sterile scalpel and then purified using the Q1Aquick Gel Extraction Kit (Qiagen, Germany) following the instructions of the manufacturer. The purified PCR product was directly used for the automated DNA sequencing using 3130 Genetic Analyzer (Applied Biosystems, Switzerland). The deduced sequence was subjected to BLAST search tool from the National Centre for Biotechnology Information, Bethesda, MD, USA (http://www.ncbi.nlm.nih.gov) to retrieve for homologous sequences deposited in GenBank (*Accession number JN034906)*.

The 16S rDNA sequence of bacteria under study was aligned with the reference sequences showing sequence homology from the NCBI database using the multiple sequence alignment programme of MEGA 6 [20]. Phylogenetic trees were constructed by distance matrix-based cluster algorithms viz. unweighted pair group method with averages (UPGMA), neighbour joining [21], maximum-likelihood [22] and maximum-parsimony [23] analyses. All positions containing gaps and missing data were eliminated from the dataset (complete deletion option). There were a total of 545 positions in the final dataset. The trees were rooted using *Bacillus* sp. HSCC 1649 T (*Accession no*. *AB045097*) as out-group. The stability of trees obtained from the above cluster analyses was assessed by using BOOTSTRAP programme in sets of 1,000 resamplings (MEGA 6).

### GC-MS analysis to identify the DBT biodegradation products

To determine the intermediates and end products of the sulfur utilization pathway, the pure culture of *Chelatococcus* sp. was grown in BSM medium containing 0.5 mM of DBT or 4- M-DBT or 4, 6 –DM-DBT (dissolved in DMF) and incubation was carried out for 96 h at 37°C on a rotary shaker at 200 rpm. A measured aliquot of the culture was withdrawn aseptically at a specific time interval and acidified to pH 2.0 by the addition of 6N HCl followed by extraction of sulfur compounds with ethyl acetate [1]. The extract was filtered through a 0.20 μm pore-size membrane filter [24]. This extract was used for the quantification of DBT or 4-M-DBT or 4, 6 –DM-DBT remained in the culture medium after inoculation with bacteria at different time intervals by using high performance liquid chromatography (Waters 515 HPLC pumps). Briefly, separation of metabolites was carried out on a Waters reversed-phase 5 μm C_18_ Nova-Pak column (3.9 mm X 150 mm) using isocratic elution under the following conditions: temperature ~25°C, mobile phase was acetonitrile: water (60: 40 v/v), flow rate was 1.0 mL min^-1^ and elution was monitored at 240 nm [25] with a Waters 2487 Dual λ absorbance detector. From a standard curve of DBT and its derivatives, the amount of residual DBT or its metabolites produced in the culture medium was determined by RP-HPLC analysis as function of retention time.

Gas chromatography-mass spectrometry (GC-MS) study was conducted to elucidate the identity of metabolites of DBT, 4-M-DBT and 4, 6 –DM-DBT produced during the process of desulfurization by bacterium under study [9]. Briefly, GC–MS (Varian, CP– 3800 GC and Saturn 2200 MS) was equipped with a quadrupole ion trap mass detector couple with CP–Sil 5 CB (0.25 mm i.d x 30 m length) capillary column, ionization energy was 70 eV, scan interval was 1.5 s and mass range was from 50–600 amu. The oven temperature was programmed at 50°C for 1.0 min with 10°C min^-1^ increment and held at 280°C for 10 min. The detector and injector temperature was kept at 280°C and Helium was used as carrier gas [9].

### Effect of 2-HBP and 2-MBP on growth performance of *Chelatococcus* sp.

To determine the viability of bacteria in presence of different concentrations of 2-MBP and/or 2-HBP (end products of DBT metabolism via 4s pathway), *Chelatococcus* sp. (1x10^6^ cfu) was grown in BSM containing 0.5 mM DBT and supplemented with different concentrations of 2-HBP, or different conentratons of 2-HBP and 2-MBP at 37°C, pH 7.0 and 200 rpm in a rotary shaker. A control was run in parallel where only 0.5 mM DBT was added in culture medium. The kinetics of bacterial growth was monitored by measuring the absorbance of culture broth samples at 600 nm.

### Isolation of bacterial intracellular proteins and identification by LC-MS/MS analysis

The pure culture of *Chelatococcus* sp. was inoculated in 100 ml of BSM medium containing 0.5 mM DBT (dissolved in DMF) and incubation was carried out for 96 h at 37°C on a rotary shaker at 200 rpm. The culture was passed through the glass wool to remove the residual DBT. The microbial culture was centrifuged at 6000 rpm for 10 min at 4°C to pellet down the cells (Heraeus Multifuge X1R Centrifuge, Thermo Scientific, USA). The supernatant was discarded and the pellet was washed three times with 20 mM Tris-HCl buffer, pH 8.0. Finally, the bacterial cells were suspended in 0.5 ml of above buffer containing cocktail of protease inhibitors (Sigma-Aldrich) to prevent the proteolysis during the subsequent steps of protein extraction. The cells were subjected to four cycles of freeze-thaw followed by 6 cycles of sonication in ice-bath for 30 sec with 1 min interval in between. The cell lysate was subjected to centrifugation at 12000 rpm for 30 min at 4°C and the supernatant containing bacterial intracellular proteins was subjected to further analysis.

The cell lysate was passed through 0.2 μm filter, and the filtrate was desalted. The protein content of the filtrate was then determined [26]. For identification of bacterial proteins by peptide mass fingerprinting (PMF) analysis our previously described procedures were followed [27, 28]. Briefly, 40 μg of bacterial proteins were reduced with 10 mM dithiothreitol at 56°C for 15 min and then alkylated with 55 mM iodoacetamide for 1 h at room temperature in the dark. This was followed by in-solution digestion with proteomics grade trypsin (50 ng/μl in 25 mM ammonium bicarbonate containing 10% acetonitrile) for 18 h at 37°C. The digested peptides were dried, reconstituted in 15 μl of the 0.1% (v/v) formic acid and then subjected to RP-nano HPLC-MS/MS analysis. The ion source was ESI (nano-spray), fragmentation modes were collision induced dissociation (y and b ions), MS scan mode was FT-ICR/Orbitrap, while MS/MS scan mode was linear ion trap. For fixed and variable modifications, carbamidomethylation of cysteine residues and oxidation of methionine residues, respectively, were selected. The data was searched against the Uniprot Swiss-Prot database (non-redundant database with reviewed proteins) and TrEMBL from National Center for Biotechnology Information (www.ncbi.nlm.nih.gov) using Peaks 7.0 software. The identified proteins were classified and grouped according to their biological function(s). To avoid multiple comparison artifacts generated through shotgun proteomics approach and to eliminate wrong identification of *Chelatococus* sp. proteins, the false discovery rate (FDR) was kept very stringent (0.8%) which was also manually varified. Further, only matching peptides and proteins showing a 10lgP value ≥ 30 and 20, respectively, were considered for identification purposes. To identify the occurrence of putative conserved domain(s), if any, in the identified desulfurizing enzymes, the MS-MS derived peptide sequences were searched in NCBI databases using BLASTp programme.

### Desulfurization of diesel fuel

The desulfurization of diesel fuel by the bacteria under study was investigated by using hydrodesulfurized (HDS) diesel fuel (total organic sulfur, 420 ppm). For the preparation of resting cells, bacterial strain was inoculated on BSM containing 0.5 mM DBT as the sole sulfur source and incubated at 37°C, 200 rpm for 96 h following the protocol described by Hirasawa et al. [29]. Desulfurization of diesel fuel by resting bacterial cells was monitored by XRF spectrometry (SRS- 53000; Siemens, Karlsruhe, Germany). Briefly, bacteria were cultured until mid-log phase and then centrifuged at 16 000x g for 10 min. The pelleted cells were washed twice with 100 mM K- phosphate buffer (pH 7.0) and then re-suspended in a fresh aliquot of the same buffer. Five mL of diesel fuel was then added to 15 mL resting cell suspension supplemented with 4% (v/v) glycerol as additional carbon source and incubated at 37°C under 200 rpm. After 24 h of incubation, the cultures were centrifuged at 16 000x g for 10 min to separate the fuel and aqueous phases. The fuel (oil) phase was analyzed for sulfur content by XRF. An aliquot of the oil phase obtained from each reaction mixture was also used for GC analysis. Un-inoculated diesel fuel sample treated under identical manner served as a control.

### Statistical analysis

Statistical analysis was done by determining the level of significance (1–5% level) between two sets of data by Student’s t-distribution (*p*-value) analysis. Both the coefficient and degree of freedom were taken into account while using table of the t-distribution [30].

## Results

### Screening, isolation and pure culture of DBT utilizing bacteria

The DBT utilizing bacterium (isolate NBTU-06) was isolated from crude petroleum-oil contaminated soil samples. DBT utilization efficacy was determined on the basis of increase in the bacterial dry biomass, protein content and bacterial cell density in the DBT containing culture medium (BSM) after 96 h of incubation (Table 1). Bacterial cell density in presence of DBT ranged from 1.4 x 10^6^–1.9 x 10^6^ mL^-1^ after 96 h of incubation at 37°C. Contrary to this, the growth of bacteria in absence of DBT (control) was found to be negligible (Table 1). On the basis of growth performance on the DBT supplemented BSM, the potent isolate was selected for further study.

**Table 1:** Bacterial growth and protein content of culture medium inoculated with *Chelatococcus* sp. in presence or absence of DBT (0.5 mM). A: growth in presence of DBT, B: growth in absence of DBT. Values are mean ± S.D of triplicate determinations. Significance of difference with respect to growth of bacteria in presence of 0.5 mM DBT.

Growth time	Cell count (number ml^-1^)		Dry biomass (mg l^-1^)		Protein content (mg ml^-1^)	
(h)	A	B	A	B	A	B
0	0.1 x 10^6^	0.1 x 10^6^	100 ± 0.005	100 ± 0.005	0.04 ± 0.002	0.04 ± 0.002
24	0.2 x 10^6^	0.02 x 10^6^*	400 ± 0.02	30 ± 0.002**	0.11 ± 0.055	0.05 ± 0.001**
48	1.4 x 10^6^	0.01 x 10^6^**	800 ± 0.04	33 ± 0.003**	0.63 ±0.032	0.04 ± 0.003**
96	1.9 x 10^6^	0.02 x 10^6^*	1560 ± 0.08	35 ± 0.004*	1.20 ± 0.06	0.05 ± 0.005**

*P<0.05

**P<0.01

### Taxonomic identification of DBT utilizing bacterial isolate

**Fig 1:**
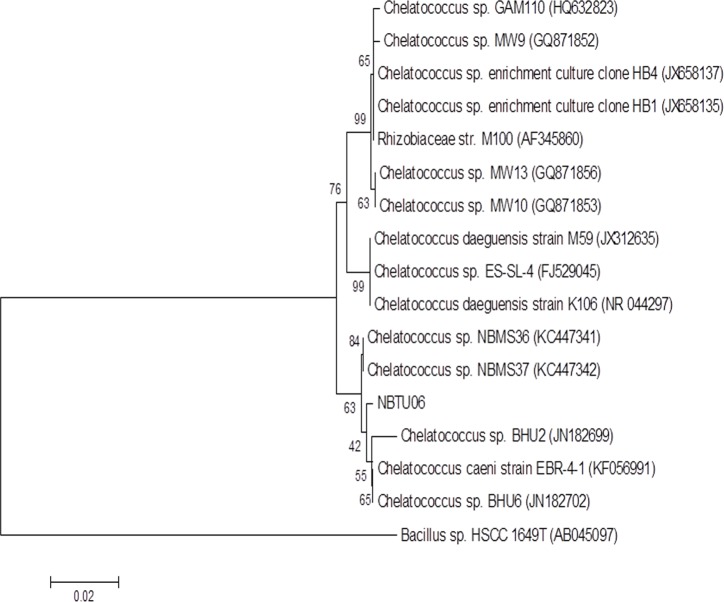
Phylogenetic relationships of isolated NBTU-06 and other closely related *Chelatococcus* species based on 16S rRNA sequencing. The tree was generated using the neighbor-joining method and the sequence from *Bacillus* sp. HSCC 1649 T (*Accession no*. *AB045097*) was considered as out-group. The data set was resampled 1,000 times by using the bootstrap option, and percentage values are given at the nodes.

The DBT utilizing isolate was an aerobic Gram-negative rod (S1 Fig) and demonstrated catalase and oxidase activities but did not hydrolyze casein. Colonies of the isolate on Luria–Bertani (LB) agar medium plates were observed as round, smooth, slightly mucoid, light yellow in color, regular and opaque under a light microscope (S2 Fig). On the basis of morphological characteristics and phenotypic properties the isolate was identified as *Chelatococcus* sp. The BLASTN search of the 16S rRNA gene sequence of the isolate revealed 95–99% similarity to *Chelatococcus* species reported in NCBI databases. The phylogenetic tree constructed from the sequence data by neighbor-joining method (Fig 1) showed the detailed evolutionary relationships between this isolate with other closely related *Chelatococcus* sp. which demonstrated a distinct phylogenetic position of this strain within the genus.

### Identification of biodesulfurization metabolites

**Fig 2:**
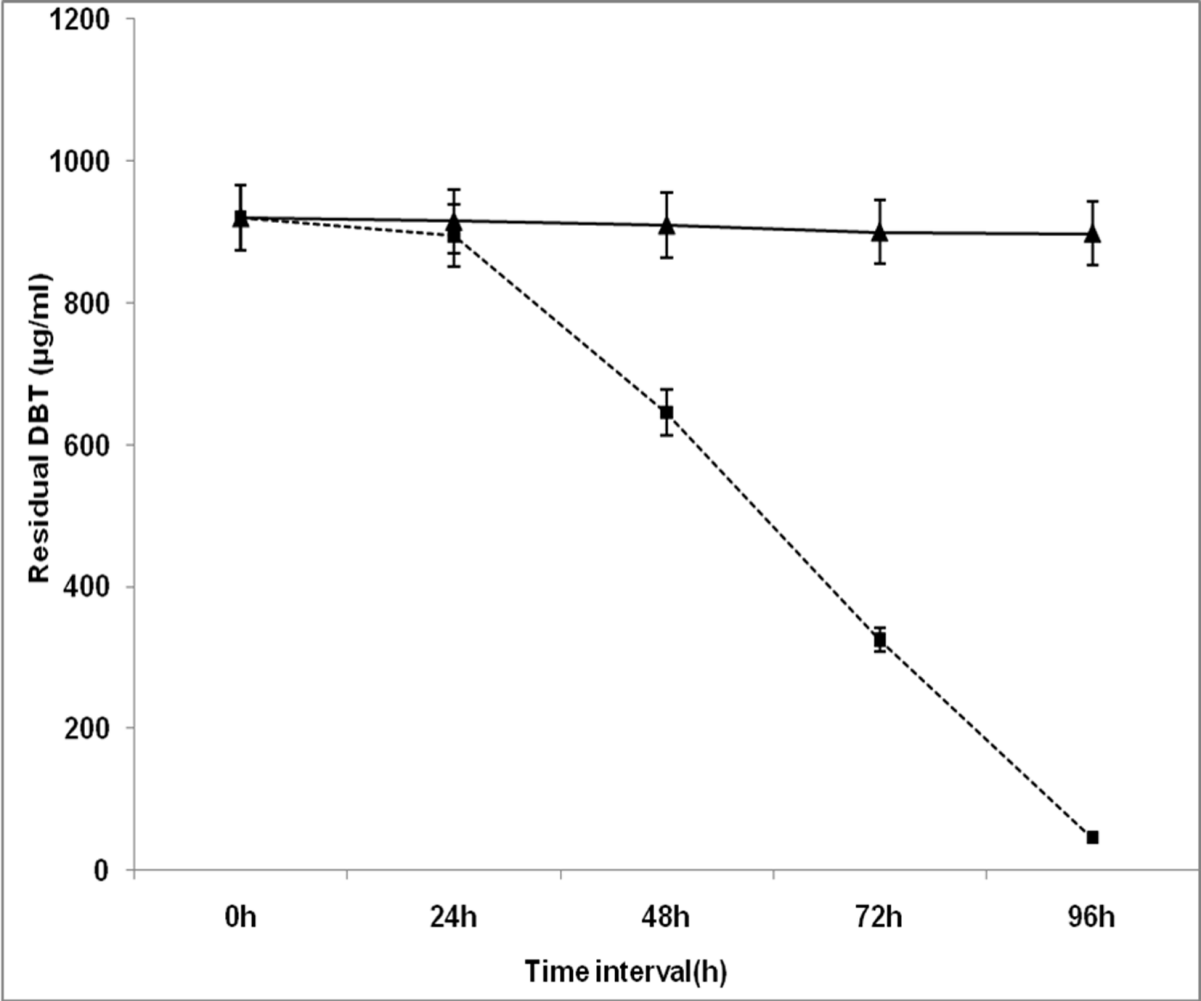
Kinetics of utilization of DBT by *Chelatococcus* sp. in liquid BSM as the sole source of sulfur. Residual DBT concentration was determined by RP-HPLC analysis of extract from the culture medium (⬛) inoculated with *Chelatococcus* sp. and uninoculated controls (▲). Values are mean ± SD of triplicate determinations.

*Chelatococcus* sp. exhibited significant growth when it was cultured in BSM containing sulfur compound such as DBT/ 4-M-DBT /4, 6-DM-DBT. The RP- HPLC analysis demonstrated that after 96 h of incubation the DBT content of the culture medium (921.30 μg ml^-1^) was decreased by *Chelatococcus* sp. to 46.07μg ml^-1^ (95% reduction) (Fig 2). In a sharp contrast, the quantity of DBT in control flask (without bacteria) was reduced to only 898.26 μg ml^-1^ (3% reduction) under identical experimental conditions. This result suggests insignificant (p > 0.05) reduction in DBT without *Chelatococcus* sp.

**Fig 3:**
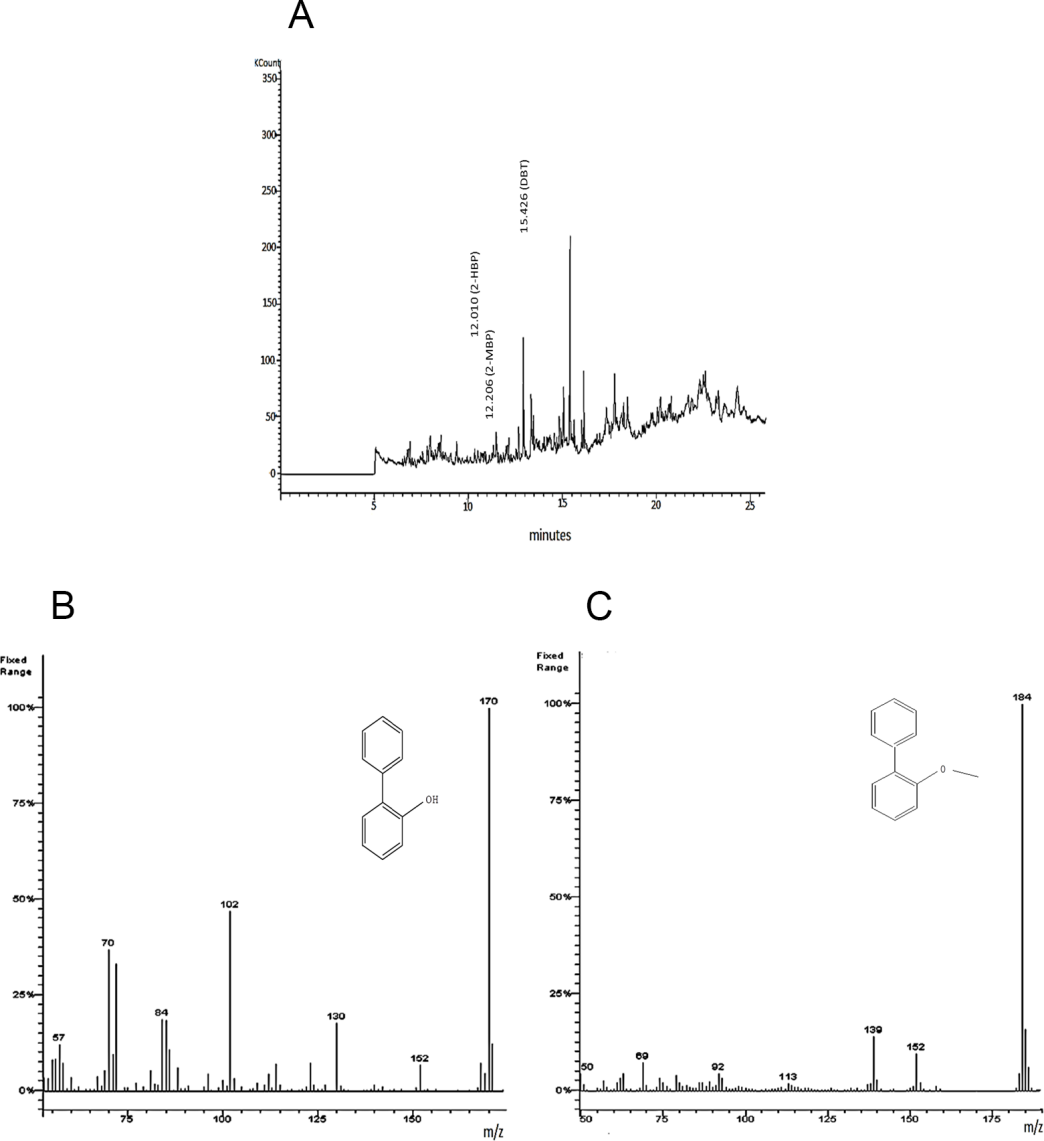
GC-MS analysis of DBT metabolites produced by C*helatococcus* sp. **A**: GC profile of the culture extract (96 h post inoculation) showing formation of 2- HBP, 2-MBP, and DBTO; **B**: Mass spectrum of 2-HBP (molecular mass, 170); **C**: Mass spectrum of 2-MBP (molecular mass, 184).

The GC and GC-MS analysis (Fig 3A–3C) of the extract of the DBT containing culture supernatant after 96 h incubation with *Chelatococcus* sp. showed appearance of several prominent peaks at different retention times. The GC chromatogram of the culture extract showed the formation of DBT metabolites like 2-HBP and 2-MBP (Fig 3A). The MS spectra showed a prominent signal at *m/z* 170 amu (MS) which signifies the occurrence of 2-HBP (Fig 3B). Although *Chelatococcus* sp. could degrade 0.5 mM DBT to 0.02 mM (determined by RP- HPLC analysis of 96 h culture supernatant) which results in ~95% reduction of initial DBT concentration; nevertheless, RP-HPLC analysis of culture extract demonstrated that the concentration of 2-HBP produced from the 0.5 mM DBT never exceed 0.02 mM. The GC-MS profile of culture supernatant identified 2- methoxy biphenyl (2-MBP) at m/z 184 as one of the end products of DBT degradation by *Chelatococcus* sp. (Fig 3C). Analysis of GC chromatogram (Fig 3A) revealed that 5.6 μM of intracellular 2-MBP was formed post 96 h of incubation with *Chelatococcus* sp. This suggests that 2-HBP was either partially converted to 2-MBP or the latter metabolite was rapidly degraded. From the GC chromatogram, the relative ratio of 2-HBP and 2-MBP formed from DBT after 96 h of growth was determined at ~4:1 (Fig 3A).

**Fig 4:**
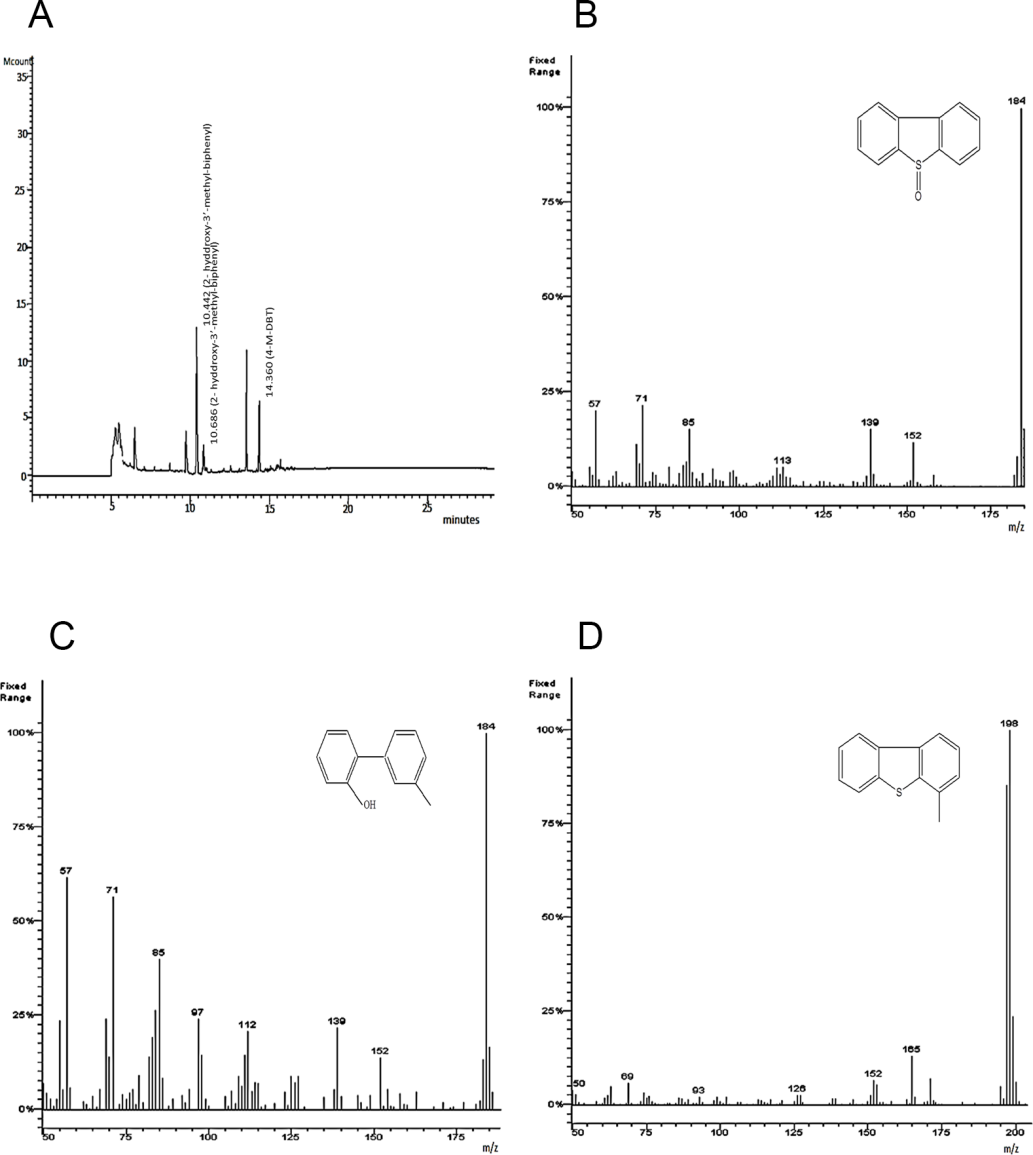
GC-MS analysis of metabolites of 4-M- DBT produced by *Chelatococcus* sp. **A**: GC chromatogram of the culture extract showing DBTO, 2-hydroxy-3’ methyl–biphenyl and 4-M- DBT; **B**: Mass spectrum of DBTO (molecular mass, 200); **C**: Mass spectrum of 2-hydroxy-3’ methyl—biphenyl (molecular mass, 184); **D**: Mass spectrum of 4-M- DBT (molecular mass, 198).

Metabolites produced by growing cells of *Chelatococcus* sp. in 4-M-DBT supplemented BSM were also identified by GC (Fig 4A) and GC–MS analysis (Fig 4B–4D). Three major peaks of metabolites with a retention time of 10.44, 10.68, 14.36 min were observed. The molecular ion at m/z 184 is in close agreement with the formation of 2-hydroxy-3-methyl-biphenyl as intermediate metabolite [11].

**Fig 5:**
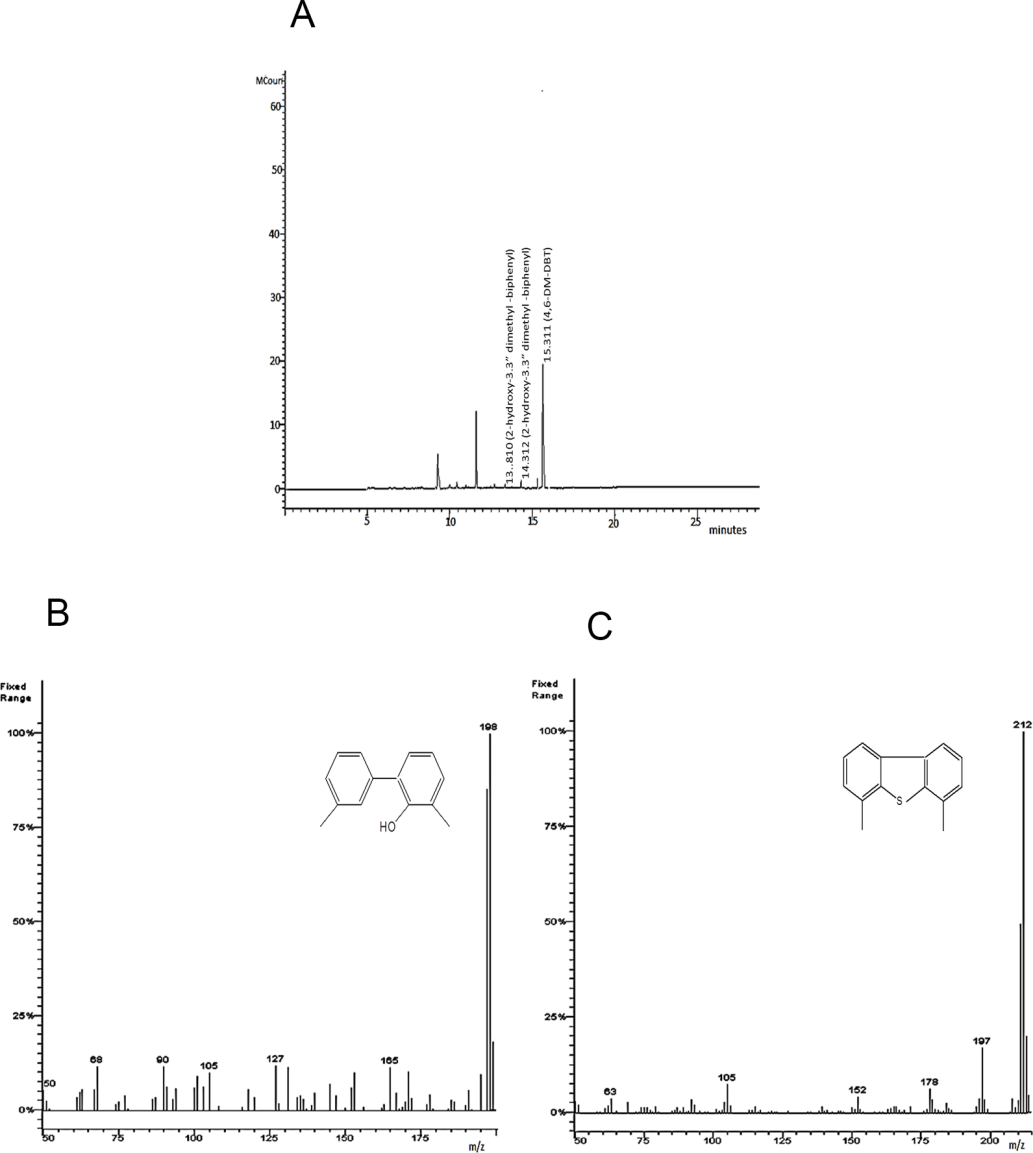
GC-MS analysis of metabolites of 4, 6 -DM- DBT produced by *Chelatococcus* sp. **A**: GC chromatogram of the culture extract showing, 2-hydroxy-3, 3’ dimethyl–biphenyl and 4, 6 -M- DBT; **B**: Mass spectrum of 2-hydroxy-3, 3’ dimethyl—biphenyl (molecular mass, 198); **C**: Mass spectrum of 4, 6-DM- DBT (molecular mass, 212).

The metabolites of 4, 6-DM-DBT desulfurization by the growing cells of *Chelatococcus* sp. as determined by GC (Fig 5A) and GC-MS analysis (Fig 5B and 5C) showed formation of 2-hydroxy –3, 3^/^- dimethyl-biphenyl (m/z 198) as the end product.

### Effect of 2-HBP and 2-MBP on growth performance of *Chelatococcus* sp.

**Fig 6:**
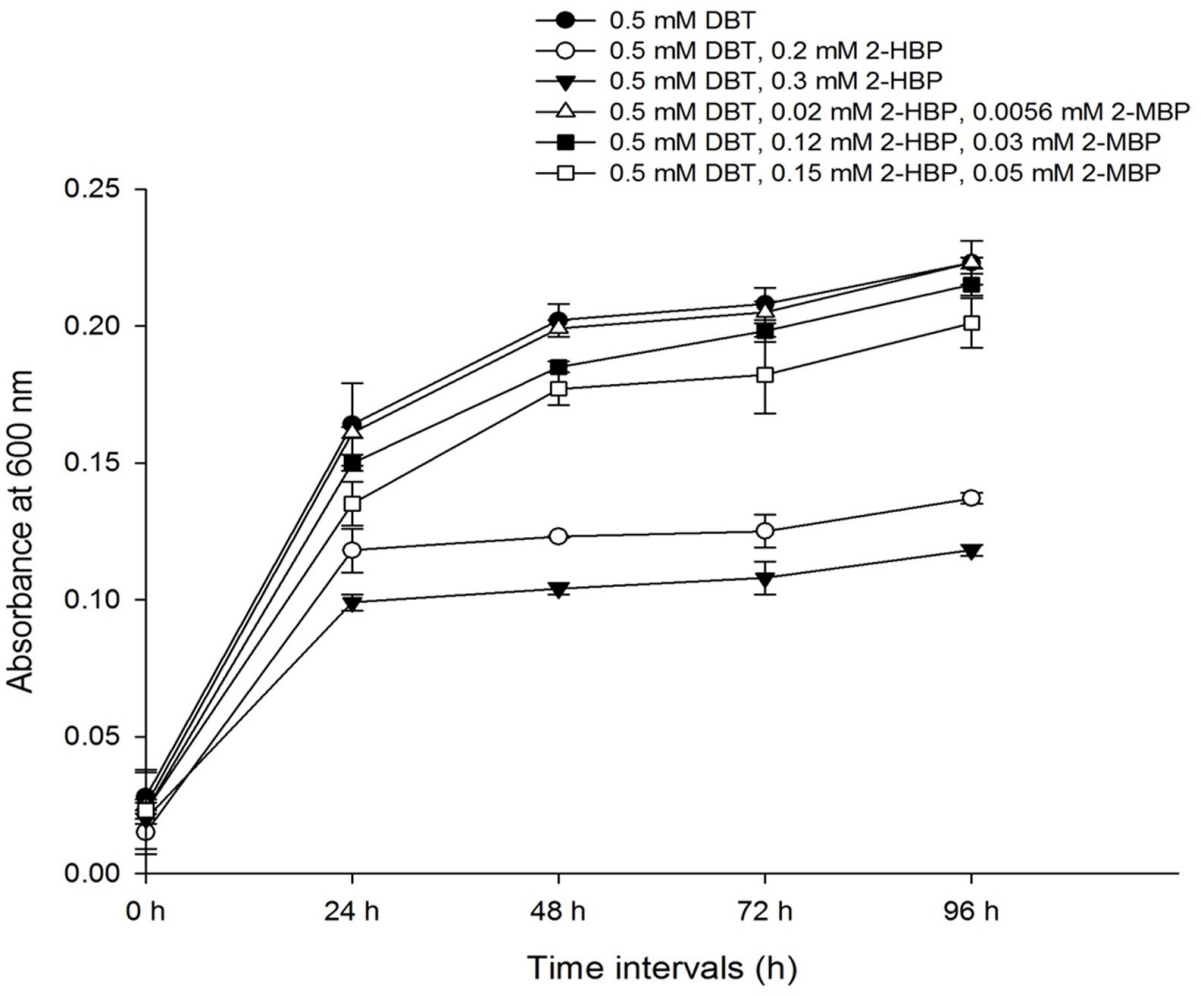
Growth performance of *Chelatococcus* sp. in presence of 0.5 mM DBT in BSM supplemented with different concentrations of 2-HBP or 2-HBP and 2-MBP after 96 h at 37°C. Values are mean ± SD of triplicate determinations.

It was observed that 2-HBP exerted a dose-dependent inhibition on growth of *Chelatococcus* sp. (Fig 6). as compared to bacterial growth in presence of 0.15 mM 2- HBP and 0.05 mM 2-MBP (Fig 6). Increasing the concentration of 2-MBP along with reduction of 2-HBP concentration resulted in further enhancement of growth of *Chelatococcus* sp. suggesting 2-MBP is significantly less toxic to bacteria (Fig 6).

### Proteomics analysis to identify the up-regulated intracellular proteins during DBT desulfurization

The desulfurization of dibenzothiophene is a complex biological process and regulated by a series of enzymes. Several intracellular proteins of *Chelatococcus* sp. having diverse biological functions were identified through LC-MS/MS analysis (S1 Table). These proteins and enzymes are associated with carbohydrate metabolism (glycolysis, gluconeogenesis, pentose phosphate shunt, glyoxylate pathway, and citric acid cycle), transport of metabolites, regulation of transcription, and translation (protein biosynthesis), stress response, biosynthesis of vitamin B6, nucleotide, fatty acids, amino acids, cell wall, quinones and porphyrins, cell division, electron transport, biological oxidation-reduction, desulfurization, and sulfur assimilation activities (S1 Table). In addition, some proteins of *Chelatococcus* sp. demonstrated similarity with eukaryotic proteins. For example, Inositol-1, 4, 5-trisphosphate 5-phosphatase 2 of *Chelatococcus* sp. demonstrated similarity with the same protein isolated from *Schizosaccharomyces pombe* (strain 972 / ATCC 24843) (S1 Table) suggesting these proteins are highly conserved during the process of evolution.

The proteomic analysis of *Chelatococcus* sp. when grown in DBT demonstrated the biosynthesis of 4S pathway desulfurizing enzymes viz. monoxygenases (DszC, DszA), desulfinase (DszB), and an NADH-dependent FMN reductase (DszD) (S1 and S2 Tables). Intracellular proteins of *Chelatococcus* sp. showing sequence homology with monooxygenase and desulfinase enzymes from different bacterial genera were identified. The alignments of MS-MS derived peptide sequences with the BDS enzymes are shown in S2 Table.

Monooxygenase, a class of enzymes, efficiently oxidizes organic substrates by introducing one oxygen atom; involved in desulfurization of dibenzothiophene are included in class C flavoprotein monooxygenases [31]. These enzymes are responsible for the oxidation of dibenzothiophene into corresponding sulphones. This group of enzymes utilizes alkanesulfonates as sulfur source in presence of reduced FMN. The proteomic analysis revealed the presence of an FMN reductase in *Chelatococcus* sp. the catalytic function of which may be assigned in obtaining sulfur from organosulfur compounds like DBT (S1 Table).

The DBT monooxygenase (DszC) enzyme catalyzes the first two steps in the DBT desulfurization process. It converts DBT to DBT sulfoxide which is then converted to DBT-sulfone. This enzyme was found to be a homotetramer with a subunit molecular mass of 50 kDa and requires FMNH_2_ as a co substrate for its activity [32]. However, Ohshiro et al. [33] reported that DBT-MO (DBT monooxygenase) isolated from *R*. *erythropolis* D-1 is a homohexamer with subunit molecular weight of 45 kDa. DBT-MO can act on various derivatives of DBT such as 2, 8- dimethyl DBT, 3, 4-benzo DBT, 4, 6- dimethyl DBT, and shows optimum activity at 40°C and pH 8.0 [34]. The MS/MS derived peptide sequences of *Chelatococcus* sp. demonstrated sequence homology with DszC monooxygenase enzymes from *Mycobacterium* sp. G3 (MW 45.37 kDa), *Rhodococcus* sp. DS-3 (MW 45.04 kDa), and *Gordonia alkanivorans* (MW ~45 kDa) (S1 and S2 Tables). Further, MS/MS derived peptide sequences of *Chelatococcus* sp TSGLLSVTVP (m/z 487.29), LSVTVPRHLGGW (m/z 682.38) and HLGGWGADWPTALEVVR (m/z 621.99), QERGELMVQISGVKAIATQ (m/z 1021.54), GELMVQISGVK (m/z 509.77), ATQAALDVTSR (m/z 378.21) showing sequence homology with DszC monooxygenase enzyme from *Mycobacterium sp*. *G3* (S1 and S2 Tables) and a peptide sequence THSLHDPVSYK (m/z 642.32) showing sequence homology with DszC monooxygenase enzymes from *Rhodococcus sp*. *DS-3* and *Gordonia alkanivorans* showed presence of putative conserved domain of acyl-CoA dehydrogenases (ACAD) superfamily. The enzymes of this superfamily are SOS adaptive response protein aidB, Naphthocyclinone hydroxylase (NcnH), and and Dibenzothiophene (DBT) desulfurization enzyme C (DszC).

DBT sulfone monooxygenase (DszA) enzyme is involved in 4S desulfuriazation pathway. The proteins of this monooxygenase family (NtaA/SnaA/SoxA/DszA) oxidizes nitriloacetate using reduced flavin mononucleotide (FMNH2) and O_2._ DszA isolated from *R*. *erythropolis* D-1 is a 97 kDa protein and comprises of two subunits each with molecular weight of ~50 kDa [35]. The DBTO_2_-MO from *R*. *erythropolis* IGTS8 requires FMNH_2_ as a coenzyme for its activity [32]. The LC-MS/MS derived peptide sequences of *Chelatococcus* sp. showed sequence similarity with DszA enzyme from *Bacillus megaterium* WSH-002 of molecular weight of 50.46 kDa (S1 and S2 Tables). Two of the MS/MS derived peptide sequences of *Chelatococcus* sp. viz. AGWNVVTSPLEGSALNYGK at m/z 981.50 and SAQGQPVVFQAGSSESGK at m/z 881.93 showing sequence similarity with DszA monooxygenase enzyme from *Bacillus megaterium* demonstrated putative conserved domain of flavin utilizing monooxygenase superfamily (S1 and S2 Tables).

HPBS desulfinase is another rate-limiting enzyme of 4S pathway which plays a crucial role in desulfurization of DBT; however, its activity has been found as the lowest in the process of desulfurization [34]. This enzyme is a monomer with subunit molecular weight of 40 kDa and shows optimum activity at 35°C [36, 37]. HPBS desulfinase does not require NADH or other co-factors for its activity and functions as an aromatic sulphinate hydrolase [38]. By LC-MS/MS analysis the tryptic peptides of *Chelatococcus* sp. showed sequence homology with DszB enzymes from *Mycobacterium* sp. G3 (MW 38.12), *R*. *erythropolis* (MW 39.07), and *Gordonia* sp. WQ-01A (MW 39.28) (S1 and S2 Tables). One of the MS/MS derived peptide sequences (NAYASVWTVSSGLVRQRPGLVQR, m/z 849.13) of *Chelatococcus* sp. showed sequence homology with DszB enzymes from *R*. *erythropolis* an*d* displayed putative conserved domain of periplasmic binding protein type-2 superfamily (S1 and S2 Tables). This group of proteins contains the ligand-binding domains of some solute binding proteins that function as initial receptors in the transport, signal transduction and channel gating. They are involved in the uptake of a variety of soluble substrates such as polysaccharides, phosphate, sulfate, lysine/arginine/ornithine, and histidine.

Flavin reductase enzyme is required by DBT monooxygenase and DBT sulfone monoooxygenase for their catalytic activity. Matsubara et al. [39] reported that the flavin reductase purified from *R*. *erythropolis* D-l is a homotetramer of molecular weight of 86 kDa with subunit molecular mass of 22 kDa. The enzyme exhibits optimum activity at 35°C and pH 6.0 [38]. Proteomic analysis showed presence of NADH-dependent FMN reductase (DszD enzyme) in *Chelatococcus* sp. (S1 Table).

By LC-MS/MS analysis, presence of another enzyme namely 5-carboxymethyl-2-hydroxymuconate semialdehyde dehydrogenase involves in 4-hydroxyphenylacetate catabolic process [40] was identified in *Chelatococcus sp*. (S1 Table). However, the exact role of this enzyme in desulfurization process is unknown.

The proteomics analysis suggested that *Chelatococcus* sp. expresses a large number of oxidoreductase enzymes when grown on DBT containing medium. The identified oxidoreductase enzymes are ferredoxin-NADP reductase (ferredoxin-NADP reductase type 2 family), putative 2-cys peroxiredoxin, succinate dehydrogenase, aldo/keto reductase, glycerol dehydrogenase, betaine aldehyde dehydrogenase, and aldehyde dehydrogenase (S1 Table). Several of the above identified enzymes showed conserved domains of respective enzyme superfamily.

### Biodesulfurization of diesel fuel

**Fig 7:**
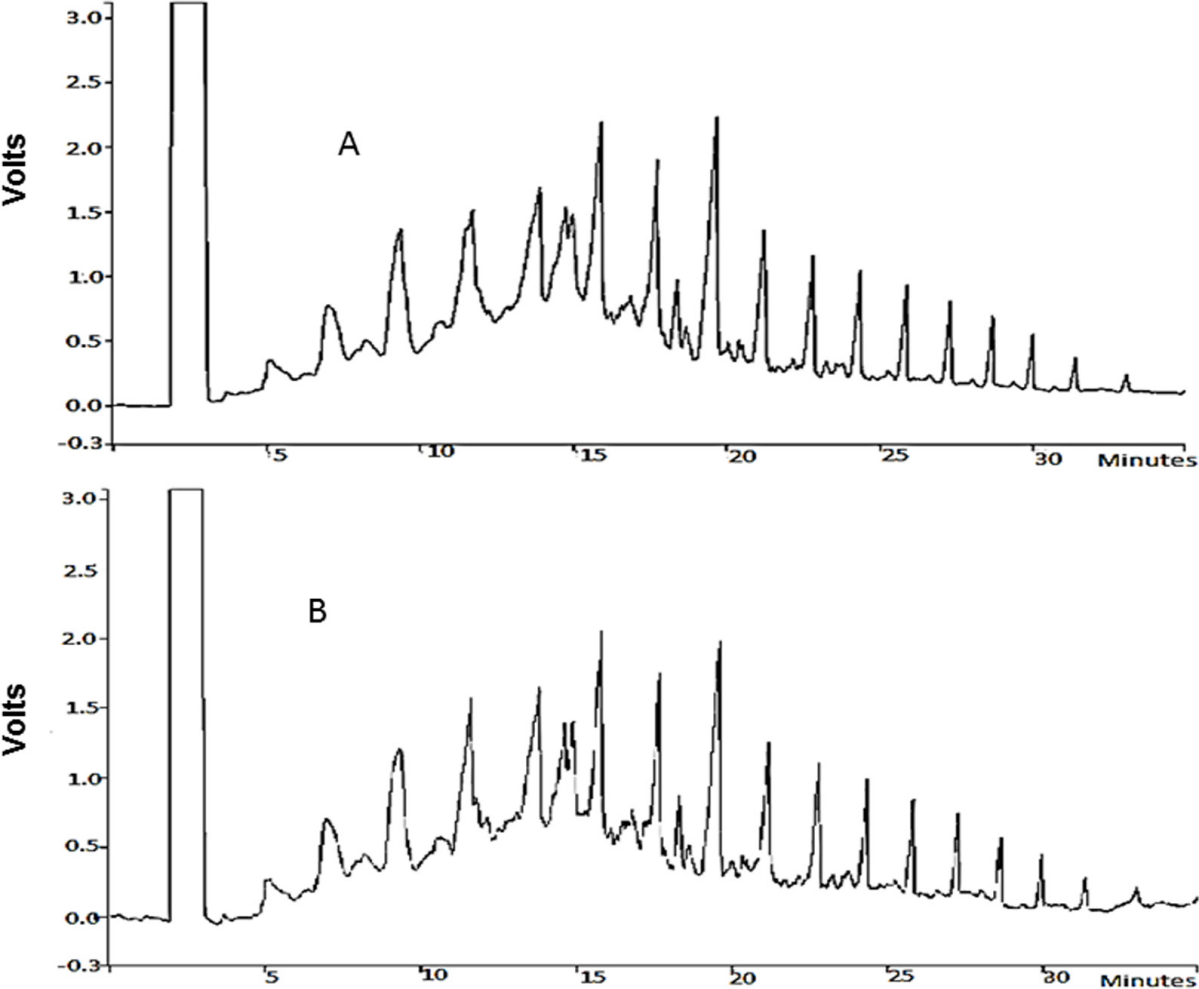
GC–FID chromatogram of desulphurization of diesel oil by *Chelatococcus* sp. **(A)** Control diesel fuel; **(B)** Diesel fuel treated with C*helatococcus* sp. for 24 h at 37°C.

*Chelatococcus* sp. was able to grow as well as desulfurize the diesel fuel which was evident from the concomitant decrease (XRF analysis) in the sulfur content of treated-fuel with respect to the control (diesel- fuel without added bacteria). The GC-FID chromatogram of the bacteria-treated diesel-fuel (Fig 7A) showed insignificant change in the resolvable peaks of this fuel, demonstrating that the bacteria utilized supplemented glycerol as source of carbon rather than the other alkane substrates of the diesel fuel. The GC-FID chromatogram therefore has provided confirmatory evidence that the quality of diesel fuel remains unaffected after treatment with *Chelatococcus* sp. (Fig 7B). The total sulfur content of the diesel fuel after 24 h treatment by 1.0 g resting cells of *Chelatococcus* sp. was reduced to 12% of its original value (before treatment).

## Discussion

The application of shotgun proteomics study in conjugation with protein database searches and protein assembly algorithms has been shown to surpass other MS-based proteomics approaches in terms of number and diversity of proteins identified and in dynamic range for detection [41]. The proteomics analysis in this study has provided an overview of expression of different cellular proteins in *Chelatococus* sp. while growing on DBT. Many of these enzymes are directly involved with desulfurization process whereas the other enzymes/proteins support growth of bacteria at an expense of DBT. To the best of our knowledge, this is the first report on proteome profiling of a bacterium while growing on DBT.

The growth of *Chelatococcus* sp. by utilizing DBT was evident from increase in bacterial biomass coupled with production of different cellular proteins and enzymes responsible for bacterial metabolism. The products of four genes viz. dsz-ABCD (*dszA*, *dszB*, *dszC*, *and dszD* genes) are shown to be involved sequentially in microbial desulfurization of DBT [16]. By LC-MS/MS analysis, two monooxygenases (DszC, DszA), a desulfinase (DszB), and an oxidoreductase (DszD) enzyme are identified in *Chelatococcus* sp. reinforcing this bacterium also follows well-known 4S pathway for DBT desulfurization [16]. The DBT monooxygenase (DszC) enzyme catalyzes the initial reactions in DBT desulfurization process that leads to formation of DBT sulfoxide (DBTO) which is rapidly converted to DBT sulfone. The second major enzyme DBT sulfone monooxygenase (DszA) involved in the process of desulfurization oxidizes DBT sulfone to HPBS. The protein sequences for DszABC genes from *Chelatococcus* sp. were found to contain the highly conserved protein motifs identified in phylogenetically diverse microbial species [42] such as the GNASSENN and FDRFWR motifs in DszC (and similar conserved protein motifs in DszA and DszB) suggesting enzymes associated with desulfurization process are highly conserved among the diverse group of bacteria.

Recently, we reported DBT desulfurization activity of *Achromobacter* sp. isolated from a petroleum-oil contaminated soil sample [9]. This bacterium is a Gram- negative rod whereas *Chelatococcus* is a Gram- negative motile bacterium. Both of these bacteria can grow at the expense of DBT; however, the growth rate of *Chelatococcus* sp. is slightly higher as compared to the growth rate of *Achromobacter* sp. in 0.5 mM DBT containing medium at 37°C [9]. Further, *Achromobacter* sp. could not grow in 4, 6- DM- DBT [9], whereas *Chelatococcus* utilized and desulfurized the above derivative of DBT under identical conditions. This suggests that although both these bacteria follow extended 4S- pathway for DBT desulfurization [9]; however they possess inherent variation in containing enzymes for desulfurization of different derivatives of DBT.

**Fig 8:**
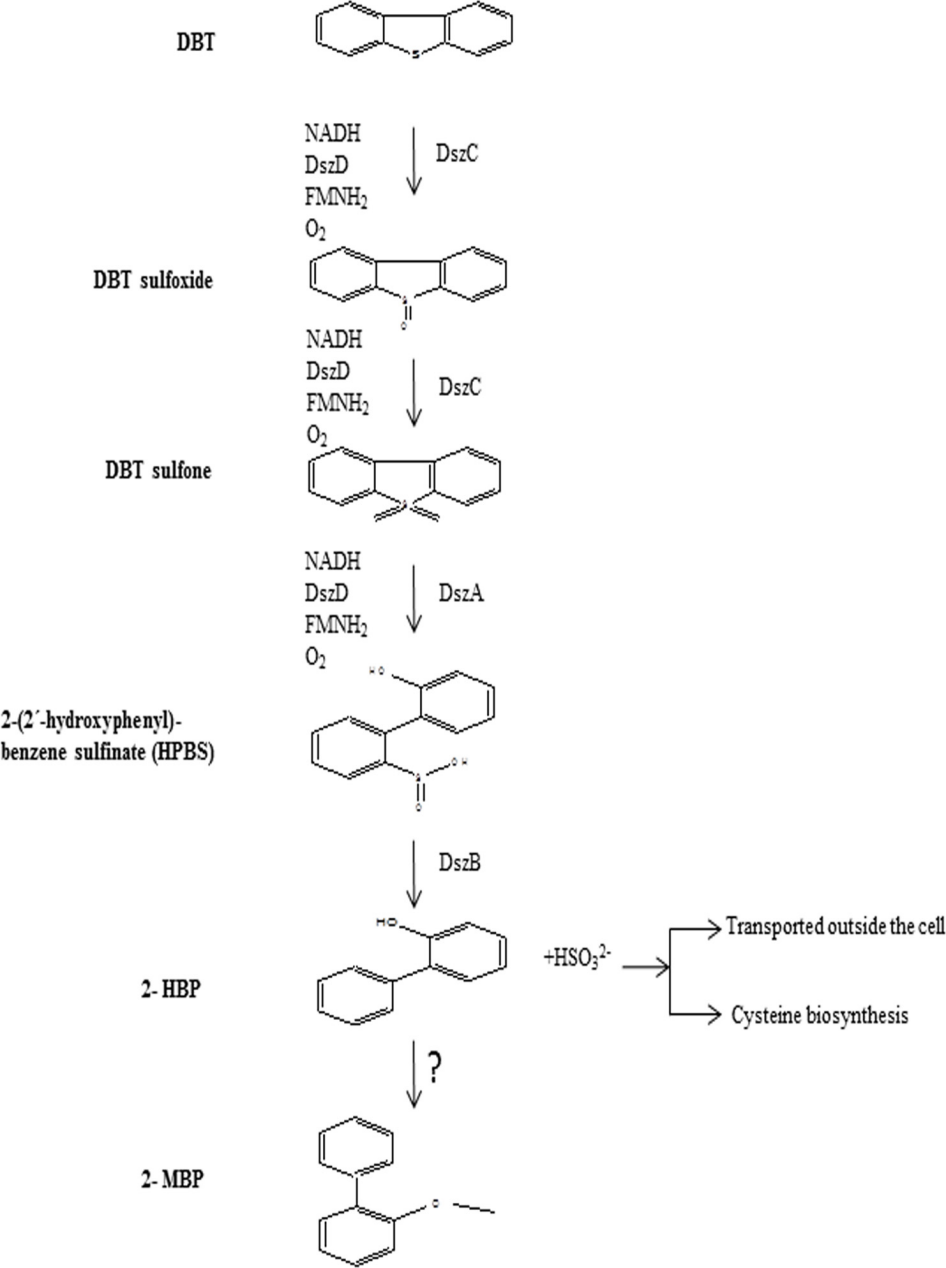
A scheme for extended 4s pathway of biocatalytic desulfurization of DBT by *Chelatococcus* sp.

Based on proteomics analysis of bacterial cell extract and GC-MS study of DBT desulfurization intermediates, a scheme for biocatalytic desulfuriation of DBT by *Chelatococcus* sp. has been proposed (Fig 8). In the first step, DBT is oxidized to DBT-sulfoxide (DBTO) and then to DBT-sulfone (DBTO_2_) by DszC monooxygenase enzyme. By GC-MS analysis, DBTO_2_ intermediate was transiently detected because it was rapidly converted to 2-(2^/^-hydroxyphenyl) benzene sulfinate (HPBS) by oxidative C-S bond cleavage in DBTO_2_ and this reaction is being catalyzed by DszA monooxygenase [38]. The HPBS was then converted into 2-hydroxybiphenyl (2-HBP) and sulfite was released by the enzyme DszB desulfinase, which is considered as the slowest step of desulfurization pathway [35]. In this 4S pathway of DBT desulfurization by *Chelatococcus sp*., the DszD oxidoreductase plays a significant role in providing the reducing equivalent (FMNH_2_) required for the function of DszC and DszA enzymes [16]. Crawford and Gupta [43] reported that cytochrome P-450 plays a key role in DBT desulfurization by the fungus *Cunninghamella elegans*. Although the exact role of cytochrome P-450 in biodesulfurization process has not been well established in bacteria however, by LC-MS/MS analysis presence of a cytochrome P-450 (Cytochrome P450 52C2) protein showing ~7% sequence similarity with cytochrome P450 from *Candida maltosa* (Yeast) was identified in *Chelatococcus* sp. (S1 Table). Therefore, it may be anticipated that synthesis of cytochrome P-450 by *Chelatococcus* sp. may also be correlated to its desulfurizing activity. Further, this proteomics analysis also suggests that cytochrome P-450 enzyme is highly conserved during the process of evolution.

Bacteria following 4S pathway of biocatalytic desulfurization release the S atom from organosulfur compounds via C-S oxidative bond cleavage. This sulfur is assimilated mainly in the form of sulfur containing amino acids to sustain the microbial growth [44]. Nevertheless, in 4S pathway the carbon frame of thiophenic compounds is not degraded and its major advantage is calorific value of the fuel after desulfurization is unaltered. Formation of 2-HBP from DBT via 4S pathway of desulfurization has been considered as the major end product of biodesulfurization [13, 16]. Therefore, it may be expected that the concentration of 2-HBP should increase concomitantly during the time course of DBT desulfurization; however, it was observed that the decrease in DBT content from the culture medium with a corresponding increase in 2-HBP production could not be correlated. Our study suggested that—2-HBP is converted to 2-MBP and some other metabolites (those could not be identified by GC-MS analysis in the present study) as end products of DBT degradation by *Chelatococcus* sp. This is accordance to our previous observation on DBT desulfurization by *Achromobactor* sp. [9] as well as reports from other laboratories demonstrating methylation at the hydroxyl group of 2-HBP leads to its conversion to 2-MBP and the concentration of 2-MBP was always less than that of 2-HBP [9, 12, 45]. This leads us to hypothesize that either 2-HBP is partially converted or 2-MBP is rapidly degraded. Nevertheless, the enzyme(s) responsible for the conversion of 2-HBP to 2-MBP remains to be identified and sequenced [9]. Due to lack of this data the presence of this enzyme in *Chelatococcus* sp. could not be ascertained by LC-MS/MS analysis.

The degradation of 2-HBP has a significant metabolic advantage for improving sulfur utilization by *Chelatococcus* sp. The present study as well as earlier studies have demonstrated that 2-HBP is toxic to bacteria because it is the major feed-back inhibitor of the *dsz-ABC* enzymes [15, 46]. Therefore once the concentration of 2-HBP reached to 0.2–0.3 mM, the biodesulfurization of DBT as well as bacterial growth could be inhibited [14, 15, 32, 46]. Therefore, methylation at the hydroxyl group 2-HBP to produce 2-MBP has significantly reduced the concentration of former to overcome the inhibitory effect of the 2-HBP on the BDS by *Chelatococcus* sp and supports its growth on DBT [9, 43]. Furthermore, 2-MBP being much less toxic compared to 2-HBP may substantially reduce the environmental pollution from the fossil fuel combustion [14, 43].

Relevantly, Li et al. [14] have reported that desulfurization of 4-M-DBT by *Mycobacterium* sp. X7B leads to the formation of 2-hydroxy-3’ methyl–biphenyl as the end product. Onaka et al. [47] reported that the desulfurization of 3-M-DBT by *R*. *erythropolis* KA 2–5–1 produces 4-methyl 2-hydroxybiphenyl as end metabolite. These results reinforce the hypothesis that the microbial desulfurizing enzyme(s) can recognize either of the two C–S bonds of DBT for the sulfur elimination processes. However, the results of microbial desulfurization of substituted DBTs like 1-M-DBT, 2-M- DBT and 4-M-DBT suggested that the cleavage of one C-S bond over the other might be preferred depending upon the position of the substituent group(s) [48]. Therefore, it may be concluded that there may be an inherent variation in utilization and desulfurization of 4, 6-DM-DBT and DBT sulfur compounds by different microbes owing to presence and expression of different desulfurization enzymes.

Although sulfur represents only 1% of total cell biomass nevertheless, it is one of the essential nutrients of the living organism for the synthesis of proteins and cofactors. The released sulfite from DBT-desulfurization by *Chelatococcus* sp *via* extended 4S pathway should be assimilated into bacterial biomass. The *R*. *erythropolis* has been shown to convert sulfite into sulfide that can be assimilated into biomass via sulfite reductase (SR) and/or sulfite oxidoreductase (SOR) pathway(s) [49]. Furthermore, it has been proposed that in addition to expression of dsz genes and requirement of additional cofactors, SR and SOR activities also play a critical role in enhancing the DBT desulfurization by *R*. *erythropolis* [49]. However, in case of *Chelatococcus* sp. the metabolism of sulfur derived from DBT via extended 4S pathway is not completely understood albeit the production of cysteine synthase enzyme in *Chelatococcus* sp. (S1 Table) suggests that synthesis of sulfur containing amino acid cysteine is a predominant mechanism of sulfur assimilation in this bacterium. The cysteine is further converted to methionine, CoA and also incorporated into proteins [50]. Moreover, synthesis of thioredoxin by *Chelatococcus* sp. also implicates its role in sulfur assimilation, protein repair and DNA synthesis [51]. Besides, enhanced synthesis of cell division proteins, transcription regulators, stress response proteins, chaperons, and activation of several metabolic enzymes by *Chelatococcus* sp. when grown in presence of DBT may also be indirectly mediated by the interaction of thioredoxin with the target proteins [51, 52].

The DBT may exert a stress on *Chelatococcus* sp. including a change in the structure-functional properties of its cell membrane that may result in overexpression of different stress proteins, antioxidants and upregulation of enzymes associated with energetic metabolism. The proteomic analysis suggested that *Chelatococcus* sp. while growing on DBT expressed enolase and ATP synthase subunits alpha and beta (S1 Table). The enolase is a key glycolytic enzyme and it is also actively involved in transportation of organic compounds across the cell membrane [53]. Therefore, the role of enolase in transportation of toxic metabolite 2-HBP / 2-MBP and/or excess sulfite to outside the cell membrane may be anticipated, and the excess energy (ATP) required for this process is provided by upregulated ATP synthase enzyme [53]. The “Major Facilitator Superfamily” (MFS) of transporter proteins identified in *Chelatococcus* sp. by LC-MS/MS analysis of its intracellular proteins may also play an important role in transporting various ions, drugs, sugar phosphates, amino acids, peptides, nucleosides, and DBT and its metabolites across the cell membrane [54]. Moreover, in order to overcome the stress exerted by the end products of DBT desulfurization (sulfite, 2-HBP and 2-MBP) *Chelatococcus* sp. also upregulates the stress proteins such as HSP-10, and HSP-60 which is accompanied by synthesis of several proteins, polypeptides and chaperons (S1 Table) all of which play a significant role in DBT desulfurization and adaptation of bacteria to DBT.

In general, both the inorganic as well as organic sulfur compounds are present in crude oil and the latter form of sulfur is represented by thiols, sulfides, disulfides and thiophenes [55]. Depending on the source of oil production, the total sulfur content of crude oil may vary from 0.03 to 7.89 wt.% [55]. DBT is regarded as the major stable, recalcitrant aromatic sulfur-containing compound of crude petroleum oil, which requires deep-desulfurization process in order to achieve the ultra-low sulfur content of the processed oil [13]. The 4S– sulfur specific pathway has been suggested as an ideal mechanism of microbial desulfurization. Therefore, intensive research has been pursued for the isolation of competent strains with potent desulfurization activity [9]. Thus for instance, Grossman et al. [56] and Li et al. [14] reported the effective removal of sulfur compounds from diesel-oil after treatment with *Mycobacterium* sp. X7B and *Rhodococcus* sp. strain ECRD-1 respectively, without compromising the fuel quality. Glucose and decane were provided as additional carbon source thereby minimizing the degradation of hydrocarbons in the diesel fuel. The *Chelatococcus* sp. has also shown significant potency to efficiently remove the sulfur compounds from the diesel fuel without compromising the quality of fuel using glycerol as the preferred carbon source for its growth. The resting cells of *Chelatococcus* sp. could reduce 12% of the total sulfur present in the HDS- treated diesel fuel and it can remove DBT from the culture medium at a rate of 75.1μmol g^-1^ h^-1^. Our previous report has shown that resting cells of *Achromobacter* sp. could reduce 7.1% of the total sulfur content of diesel fuel [9]. Mohamed et al. [16] reported that the DBT consumption rate of bacterial isolate SA11 was 11 μmol G DCW^-1^ h^-1^ at the early exponential phase of bacterial growth. Furthermore, the resting cells of SA11 exhibited 10% reduction in total sulfur present in heavy crude oil and 18% reduction in total sulfur present in the hexane-soluble fraction of the heavy crude oil [16]. Therefore, taken together it may be anticipated that *Chelatococcus* sp. is a very promising candidate for ecobenevolent microbial desulfurization of diesel fuel.

## Supporting Information

S1 Table List of intracellular proteins of *Chelatococcus* sp. identified by LC-MS/MS analysis and classified based on diverse biological functions. (DOC)

S2 Table The alignments of MS-MS derived peptide sequences of *Chelatococcus sp*. with the BDS enzymes reported in the databases. The peptides identified by MS/MS sequencing in this proteome using PEAKS 7.0 software are shown in bold and putative conserved domains have been underlined.
(DOC)

S1 Fig Gram’s staining light micrograph of *Chelatococcus* sp. under 100X magnification. (TIF)

S2 Fig Pure culture of *Chelatococcus* sp. grown on lysogeny agar (LB) plates. (TIF)
