# Supplementary material for: Proteomics and Metabolomics Analyses to Elucidate the Desulfurization Pathway of Chelatococcus sp
Source: PLoS One. 2016 Apr 21;11(4):e0153547. doi: 10.1371/journal.pone.0153547 (PMC4839641; doi:10.1371/journal.pone.0153547)
Supplement: S1 Table — (DOC) [file pone.0153547.s001.doc]

S1 Table. List of intracellular proteins of *Chelatococcus* sp. identified by LC-MS/MS analysis and classified based on diverse biological functions.

| **Accession No.** | **Score** | **Coverage (%)** | **Unique Peptides** | **MS-MS peptides** | **Avg. Mass** | **Matching Protein** | **Source of Protein**  **(Bacterial sp./ Eukaryotic organisms)** | **Protein family** | **Biological Significance** |
| --- | --- | --- | --- | --- | --- | --- | --- | --- | --- |
| G2RXU1 | 185.36 | 36.25 | 3 | 5 | 26.90 | Triosephosphate isomerase | *Bacillus megaterium WSH-002* | Triosephosphate isomerase family | Glycolytic process, Gluconeogenesis, pentose phosphate shunt |
| P23722 | 84.63 | 13.13 | 1 | 2 | 35.88 | Glyceraldehyde-3-phosphate dehydrogenase | *Bacillus megaterium (strain DSM 319)* | Glyceraldehyde-3-phosphate dehydrogenase family | Glycolysis, Oxidoreductase |
| P94453 | 78.88 | 18.12 | 1 | 4 | 30.80 | Fructose-bisphosphate aldolase | *Geobacillus stearothermophilus (Bacillus stearothermophilus)* | Class II fructose-bisphosphate aldolase family | Glycolytic process |
| P41579 | 32.66 | 6.74 | 0 | 2 | 48.85 | 6-phosphogluconate dehydrogenase, decarboxylating | *Shigella dysenteriae* | 6-phosphogluconate dehydrogenase family | Pentose phosphate shunt, gluconate utilization |
| D7UWH0 | 137.83 | 7.67 | 0 | 2 | 46.19 | Enolase | *Listeria grayi DSM 20601* | Enolase family | Glycolytic process |
| A0A0A8JDW5 | 137.83 | 7.66 | 0 | 2 | 46.71 |  | *Bacillus sp. (strain OxB-1)* |  |  |
| R9C685 | 135.55 | 12.06 | 0 | 3 | 46.39 |  | *Bacillus nealsonii AAU1* |  |  |
| A0A0C2RYG8 | 100.97 | 8.64 | 0 | 2 | 51.16 | Fumarate hydratase class II | *Jeotgalibacillus campisalis* | Class-II fumarase/aspartase family | TCA Cycle |
| G9QIV1 | 100.97 | 8.75 | 0 | 2 | 50.74 |  | *Bacillus smithii 7_3_47FAA* |  |  |
| I3E665 | 100.97 | 8.71 | 0 | 2 | 50.74 |  | *Bacillus methanolicus PB1* |  |  |
| W1SG14 | 100.97 | 8.71 | 0 | 2 | 50.42 |  | *Bacillus vireti LMG 21834* |  |  |
| P39120 | 96.60 | 10.48 | 1 | 2 | 41.70 | Citrate synthase | *Bacillus subtilis (strain 168)* | Citrate synthase family |  |
| A0A090KU98 | 91.56 | 12.13 | 0 | 3 | 41.30 |  | *Bacillus thermoamylovorans* |  |  |
| Q8EPE0 | 91.56 | 9.92 | 0 | 2 | 41.97 |  | *Oceanobacillus iheyensis (strain DSM 14371 / JCM 11309 / KCTC 3954 / HTE831)* |  |  |
| G2RJT2 | 84.74 | 10.43 | 0 | 4 | 72.50 | Transketolase | *Bacillus megaterium WSH-002* | Transketolase family | Pentose phosphate pathway |
| G2RSA8 | 81.40 | 22.11 | 1 | 4 | 30.43 | Fructose-1,6-bisphosphate aldolase, class II | *Bacillus megaterium WSH-002* | Class II fructose-1,6 bisphosphate aldolase family | Glycolytic process |
| B9CTR4 | 76.24 | 10.68 | 0 | 3 | 49.60 | Dihydrolipoyl dehydrogenase | *Staphylococcus capitis SK14* | Class-I pyridine nucleotide-disulfide oxidoreductase family | Glycolytic process, Cell redox homeostasis |
| Q5HGY8 | 84.65 | 12.18 | 0 | 4 | 49.42 |  | *Staphylococcus aureus (strain COL)* |  |  |
| D5DBS9 | 76.24 | 15.96 | 0 | 5 | 49.28 |  | *Bacillus megaterium (strain DSM 319)* |  |  |
| A0A033V5I0 | 76.24 | 12.39 | 0 | 4 | 48.94 |  | *Staphylococcus aureus C0673* |  |  |
| G2RQL2 | 67.49 | 9.2 | 0 | 3 | 57.12 | Glucose-6-phosphate 1-dehydrogenase | *Bacillus megaterium WSH-002* | Glucose-6-phosphate dehydrogenase family | Carbohydrate Metabolism, pentose phosphate shunt |
| A0A0D3VBA1 | 80.80 | 13.92 | 0 | 4 | 47.70 | Isocitrate dehydrogenase | *Paenibacillus sp. IHBB 10380* | Isocitrate and isopropylmalate dehydrogenases family | Glyoxylate bypass |
| G2RVW4 | 66.69 | 15.19 | 0 | 5 | 46.68 |  | *Bacillus megaterium WSH-002* |  |  |
| K9LF51 | 59.07 | 9.5 | 0 | 4 | 24.30 |  | *Exiguobacterium sp. 11_5m1* |  |  |
| P39126 | 45.08 | 8.27 | 0 | 3 | 46.39 |  | *Bacillus subtilis (strain 168)* |  |  |
| D5DMW6 | 47.13 | 8.19 | 0 | 3 | 61.75 | Pyruvate kinase | *Bacillus megaterium (strain DSM 319)* | Pyruvate kinase family | Glycolytic process |
| D5DMW0 | 44.88 | 9.62 | 0 | 2 | 33.30 | Malate dehydrogenase | *Bacillus megaterium (strain DSM 319)* | MDH type 3 family | TCA Cycle |
| A0A098FDJ2 | 43.82 | 6.22 | 0 | 2 | 41.46 | Succinyl-CoA ligase [ADP-forming] subunit beta | *Bacillus simplex* | Succinate/malate CoA ligase beta subunit family |  |
| P17674 | 170.56 | 22.51 | 2 | 9 | 54.57 | ATP synthase subunit alpha | *Bacillus megaterium (strain ATCC 12872 / QMB1551)* | ATPase alpha/beta chains family | ATP synthesis/transport/ ATP hydrolysis coupled proton transport |
| Q71WP7 | 43.64 | 10.32 | 0 | 5 | 55.04 |  | *Listeria monocytogenes serotype 4b (strain F2365)* |  |  |
| A8FIB4 | 43.64 | 11.16 | 0 | 5 | 54.61 |  | *Bacillus pumilus (strain SAFR-032)* |  |  |
| M5JG85 | 90.64 | 15.01 | 0 | 4 | 51.92 | ATP synthase subunit beta | *Anoxybacillus flavithermus TNO-09.006* |  |  |
| A9VSA3 | 65.90 | 14.5 | 1 | 4 | 51.09 |  | *Bacillus weihenstephanensis (strain KBAB4)* |  |  |
| B7GMF3 | 86.37 | 15.01 | 0 | 4 | 51.92 |  | *Anoxybacillus flavithermus (strain DSM 21510 / WK1)* |  |  |
| P12698 | 86.37 | 21.14 | 0 | 7 | 51.51 |  | *Bacillus megaterium (strain ATCC 12872 / QMB1551)* |  |  |
| G2RX21 | 76.06 | 9.95 | 0 | 4 | 63.62 | Phosphoenolpyruvate-protein phosphotransferase | *Bacillus megaterium WSH-002* | PEP-utilizing enzyme family | Sugar transport, phosphoenolpyruvate-protein phosphotransferase activity |
| K2G7M6 | 154.48 | 8.53 | 1 | 4 | 76.68 | Elongation factor G | *Salimicrobium jeotgali* | Classic translation factor GTPase family | Protein biosynthesis |
| A0A063Z0X2 | 154.22 | 8.53 | 0 | 4 | 76.44 |  | *Bacillus pumilus (Bacillus mesentericus)* |  |  |
| Q8DVV4 | 151.61 | 9.52 | 1 | 4 | 76.62 |  | *Streptococcus mutans serotype c (strain ATCC 700610 / UA159)* |  |  |
| I3E0K1 | 147.75 | 11.42 | 0 | 5 | 76.74 |  | *Bacillus methanolicus PB1* |  |  |
| A0A090IQR0 | 136.89 | 12.72 | 0 | 6 | 76.78 |  | *Bacillus thermoamylovorans* |  |  |
| E5WSX0 | 119.31 | 9.54 | 1 | 5 | 76.53 |  | *Bacillus sp. 2_A_57_CT2* |  |  |
| F3B2T6 | 81.31 | 7.77 | 0 | 2 | 44.05 | Elongation factor Tu | *Lachnospiraceae oral taxon 107 str. F0167* |  |  |
| A7I3U7 | 46.36 | 7.27 | 1 | 2 | 43.63 |  | *Campylobacter hominis (strain ATCC BAA-381 / LMG 19568 / NCTC 13146 / CH001A)* |  |  |
| 518231351 | 34.94 | 3.79 | 1 | 1 | 43.36 |  | *Chelatococcus sp. GW1* |  |  |
| Q8ES77 | 33.09 | 7.77 | 0 | 2 | 53.88 | Aspartyl/glutamyl-tRNA(Asn/Gln) amidotransferase subunit B | *Oceanobacillus iheyensis (strain DSM 14371 / JCM 11309 / KCTC 3954 / HTE831)* | GatB/GatE family |  |
| A0A0C1BFX7 | 126.24 | 30.77 | 0 | 4 | 17.20 | Transcription elongation factor GreA | *Bacillus thermotolerans* | GreA/GreB family |  |
| G2RXY1 | 102.87 | 25.32 | 1 | 3 | 17.47 |  | *Bacillus megaterium WSH-002* |  |  |
| G2RJK1 | 115.02 | 30.38 | 2 | 7 | 32.09 | Elongation factor Ts | *Bacillus megaterium WSH-002* | EF-Ts family |  |
| Q5L420 | 112.89 | 12.02 | 1 | 2 | 24.92 | 50S ribosomal protein L1 | *Geobacillus kaustophilus (strain HTA426)* | Ribosomal protein L1P family | Translation regulation |
| G2RS19 | 76.48 | 18.18 | 1 | 3 | 24.89 |  | *Bacillus megaterium WSH-002* |  |  |
| A0A0B0HLY0 | 75.7 | 9.27 | 0 | 2 | 40.15 | Methionine--tRNA ligase | *Anoxybacillus sp. BCO1* | Class-I aminoacyl-tRNA synthetase family | Protein Biosynthesis |
| S0RYJ3 | 43.97 | 3.35 | 0 | 2 | 95.71 | Leucine--tRNA ligase | *Enterococcus avium ATCC 14025* |  |  |
| Q9K7S8 | 43.97 | 3.85 | 0 | 2 | 92.01 |  | *Bacillus halodurans (strain ATCC BAA-125 / DSM 18197 / FERM 7344 / JCM 9153 / C-125)* |  |  |
| U5L4L8 | 65.74 | 38.02 | 0 | 6 | 12.64 | 50S ribosomal protein L7/L12 | *Bacillus infantis NRRL B-14911* | Ribosomal protein L7/L12P family | Translation, ribosomal protein |
| G4D102 | 64.97 | 11.11 | 0 | 2 | 12.92 |  | *Peptoniphilus indolicus ATCC 29427* |  |  |
| P42919 | 51.35 | 12.64 | 1 | 2 | 30.26 | 50S ribosomal protein L2 | *Bacillus subtilis (strain 168)* | Ribosomal protein L2P family | Translation, ribosomal protein, structural constituent of protein |
| C5D3S0 | 51.26 | 14.13 | 0 | 5 | 30.27 |  | *Geobacillus sp. (strain WCH70)* |  |  |
| D7CJM0 | 51.26 | 7.3 | 0 | 3 | 29.74 |  | *Syntrophothermus lipocalidus (strain DSM 12680 / TGB-C1)* |  |  |
| G2RQE2 | 51.26 | 13.77 | 0 | 4 | 30.21 |  | *Bacillus megaterium WSH-002* |  |  |
| R6AQE0 | 51.26 | 16.25 | 0 | 4 | 30.22 |  | *Clostridium sp. CAG:138* |  |  |
| J8Q902 | 49.97 | 22.70 | 0 | 2 | 15.0 | 50S ribosomal protein L11 | *Bacillus alcalophilus ATCC 27647* | Ribosomal protein L11P family | Translation/ Structural constituent of ribosome |
| C5D3Q4 | 51.83 | 39.72 | 0 | 4 | 14.98 |  | *Geobacillus sp. (strain WCH70)* |  |  |
| D5DL32 | 52.23 | 19.31 | 0 | 6 | 41.51 | 30S ribosomal protein S1 | *Bacillus megaterium (strain DSM 319)* | Ribosomal protein S1P family |  |
| A0A0A8X3Q4 | 48.75 | 11.35 | 0 | 3 | 42.05 | SSU ribosomal protein S1p | *Bacillus selenatarsenatis SF-1* |  |  |
| P0A356 | 134.97 | 50.00 | 1 | 3 | 7.26 | Cold shock-like protein CspLA | *Listeria innocua serovar 6a (strain CLIP 11262)* | CSD family | Transcription regulation, Stress response |
| A8FD81 | 82.77 | 11.20 | 0 | 4 | 29.03 | GTP-sensing transcriptional pleiotropic repressor CodY | *Bacillus pumilus (strain SAFR-032)* | CodY family | Transcription, Transcription regulation |
| A0A098EWR1 | 78.60 | 11.97 | 1 | 3 | 28.99 |  | *Bacillus sp. B-jedd* |  |  |
| G2RQB8 | 45.51 | 10.83 | 0 | 3 | 35.08 | DNA-directed RNA polymerase subunit alpha | *Bacillus megaterium WSH-002* | RNA polymerase alpha chain family | Transcription, Transcription regulation |
| G2RRN6 | 144.24 | 40 | 1 | 2 | 7.19 | Cold shock-like protein cspB | *Bacillus megaterium WSH-002* | CSD family | Transcription regulation |
| A8MGJ3 | 51.48 | 3.67 | 0 | 2 | 72.99 | Chaperone protein HtpG | *Alkaliphilus oremlandii (strain OhILAs) (Clostridium oremlandii (strain OhILAs))* | Heat shock protein 90 family | Stress response, chaperone, protein folding |
| B2A875 | 51.48 | 3.99 | 0 | 2 | 73.01 |  | *Natranaerobius thermophilus (strain ATCC BAA-1301 / DSM 18059 / JW/NM-WN-LF)* |  |  |
| A0A0B6A6A3 | 108.74 | 10.08 | 0 | 5 | 65.21 | Chaperone protein DnaK | *Bacillus megaterium NBRC 15308 = ATCC 14581* | Heat shock protein 70 family |  |
| C2WD30 | 36.15 | 15.72 | 0 | 2 | 17.51 | Universal stress protein | *Bacillus cereus Rock3-44* | Universal stress protein A family | Stress response |
| A0A0C1B7S3 | 124.36 | 11.83 | 0 | 5 | 57.30 | 60 kDa chaperonin | *Bacillus thermotolerans* | Chaperonin (HSP60) family | Chaperone, protein folding |
| A0A084GQD4 | 134.07 | 10.52 | 0 | 5 | 57.15 |  | *Bacillus indicus LMG 22858* |  |  |
| A7Z207 | 127.86 | 11.95 | 0 | 5 | 57.38 |  | *Bacillus amyloliquefaciens subsp. plantarum (strain DSM 23117 / BGSC 10A6 / FZB42)* |  |  |
| D5DWV7 | 98.70 | 5.71 | 0 | 2 | 57.16 |  | *Bacillus megaterium (strain ATCC 12872 / QMB1551)* |  |  |
| A0A090J4X8 | 97.74 | 8.91 | 1 | 5 | 57.18 |  | *Bacillus thermoamylovorans* |  |  |
| Q38YR7 | 86.23 | 4.07 | 0 | 2 | 57.24 |  | *Lactobacillus sakei subsp. sakei (strain 23K)* |  |  |
| A8MJJ7 | 86.23 | 5.73 | 0 | 3 | 57.57 |  | *Alkaliphilus oremlandii (strain OhILAs) (Clostridium oremlandii (strain OhILAs))* |  |  |
| Q5L3E6 | 60.9 | 5.95 | 0 | 3 | 57.27 |  | *Geobacillus kaustophilus (strain HTA426)* |  |  |
| Q65MZ9 | 65.16 | 40.43 | 0 | 3 | 10.11 | 10 kDa chaperonin | *Bacillus licheniformis (strain ATCC 14580 / DSM 13 / JCM 2505 / NBRC 12200 / NCIMB 9375 / NRRL NRS-1264 / Gibson 46)* | GroES chaperonin family |  |
| G2RT82 | 140.31 | 11.8 | 0 | 3 | 50.46 | Monooxygenase, NtaA/SnaA/SoxA/DszA family protein | *Bacillus megaterium WSH-002* | Monooxygenase family | Desulfurizing activity,  Monooxygenase activity |
| BAC41358 | 59.78 | 11 | 4 | 4 | 38.12 | DBTO2 HBPS desulfinase DszB | *Mycobacterium sp. G3* | Desulfinase family | Desulfurization activity,  Desulfinase activity |
| AAP80183 | 56.34 | 18 | 5 | 6 | 39.07 | DszB (plasmid) | *Rhodococcus erythropolis* |  |  |
| ABE02291 | 26.98 | 8 | 1 | 2 | 39.28 | Mutant HPBS desulfinase (dszB) | *Gordonia sp. WQ-01A* |  |  |
| BAC41359 | 75.9 | 29 | 16 | 16 | 45.37 | DBT monooxygenase DszC | *Mycobacterium sp. G3* | Monooxygenase family | Desulfurization activity, Monooxygenase activity |
| ABE26646 | 47.37 | 15 | 1 | 5 | 45.04 | DszC | *Rhodococcus sp. DS-3* |  |  |
| AAU14819 | 47.34 | 20 | 1 | 6 | 44.88 |  | *Gordonia alkanivorans* |  |  |
| AAT78718 | 25.06 | 4 | 1 | 2 | 45.09 | DBT monooxygenase | *Gordonia alkanivorans* |  |  |
| H0RG75 | 40.53 | 17 | 3 | 3 | 19.22 | NADH-dependent FMN reductase DszD | *Gordonia polyisoprenivorans* | Oxidoreductase family | Oxidoreductase activity |
| A7WYT1 | 72.29 | 9.15 | 0 | 3 | 31.97 | Pyridoxal 5'-phosphate synthase subunit PdxS | *Staphylococcus aureus (strain Mu3 / ATCC 700698)* | PdxS/SNZ family | pyridoxal phosphate biosynthetic process |
| B9E835 | 67.57 | 8.12 | 0 | 3 | 49.84 | Adenylosuccinate lyase | *Macrococcus caseolyticus (strain JCSC5402)* | Lyase 1 family | Nucleotide biosynthesis (purine) |
| W7CJ75 | 67.57 | 10 | 0 | 4 | 49.18 |  | *Listeria weihenstephanensis FSL R9-0317* |  |  |
| A0A031IBB5 | 58.77 | 5.58 | 0 | 2 | 47.44 | Adenylosuccinate synthetase | *Exiguobacterium sp. RIT341* | Adenylosuccinate synthetase family |  |
| Q5KU76 | 58.77 | 9.81 | 0 | 3 | 47.36 |  | *Geobacillus kaustophilus (strain HTA426)* |  |  |
| A0A0D8YT76 | 84.38 | 11.63 | 0 | 2 | 27.70 | Enoyl-ACP reductase | *Bacillus licheniformis* | Short-chain dehydrogenases  /reductases (SDR) family | Fatty acid biosynthetic process |
| R9CBG1 | 113.73 | 17.58 | 0 | 2 | 19.41 | Cell-division initiation protein DivIVA | *Bacillus nealsonii AAU1* | FtsZ family | Cell cycle, cell division |
| P17865 | 110.60 | 15.71 | 4 | 6 | 40.37 | Cell division protein FtsZ | *Bacillus subtilis (strain 168)* |  |  |
| A0A0D0R273 | 92.17 | 22.52 | 0 | 6 | 39.23 |  | *Anoxybacillus thermarum* |  |  |
| N9VA73 | 63.49 | 3.49 | 0 | 2 | 40.23 |  | *Mycoplasma auris 15026* |  |  |
| A0A0D0QXI0 | 57.89 | 17.65 | 0 | 2 | 30.09 | 1,4-dihydroxy-2-naphthoyl-CoA synthase | *Anoxybacillus thermarum* | Enoyl-CoA hydratase/isomerase family | Menaquinone biosynthesis |
| D7CPM1 | 124.42 | 6.69 | 0 | 3 | 73.18 | Flagellin domain protein | *Syntrophothermus lipocalidus (strain DSM 12680 / TGB-C1)* | Bacterial flagellin family | Cell motility |
| A0A087LGX3 | 124.42 | 13.53 | 0 | 3 | 46.23 | Flagellin | *Geobacillus stearothermophilus (Bacillus stearothermophilus)* |  |  |
| G2TM63 | 449.96 | 26.67 | 0 | 3 | 9.67 | Histone family protein DNA-binding protein | *Bacillus coagulans 36D1* | Bacterial histone-like protein family | DNA condensation |
| G2RJ37 | 99.67 | 57 | 0 | 6 | 11.01 | DNA-binding protein HU-alpha (Histone-like protein) | *Bacillus megaterium WSH-002* |  |  |
| G2RQ60 | 63.33 | 48.89 | 2 | 4 | 9.61 |  | *Bacillus megaterium WSH-002* |  |  |
| L5N3V3 | 62.22 | 26.67 | 0 | 3 | 9.77 | DNA-binding protein HU | *Halobacillus sp. BAB-2008* |  |  |
| V6IYC8 | 62.22 | 26.67 | 0 | 3 | 9.57 | Transcriptional regulator | *Sporolactobacillus laevolacticus DSM 442* |  |  |
| A6CJQ5 | 56.07 | 18.68 | 0 | 2 | 9.88 | Non-specific DNA-binding protein | *Bacillus sp. SG-1* |  |  |
| P08821 | 50.04 | 42.39 | 1 | 4 | 9.88 | DNA-binding protein HU 1 | *Bacillus subtilis (strain 168)* |  |  |
| D3FQJ8 | 101.53 | 11.15 | 0 | 3 | 60.79 | Urocanate hydratase | *Bacillus pseudofirmus (strain OF4)* | Urocanase family | Histidine metabolism |
| A0A098FC26 | 101.53 | 14.87 | 0 | 5 | 60.99 |  | *Bacillus simplex* |  |  |
| Q8EKJ5 | 64.48 | 4.32 | 0 | 2 | 60.35 |  | *Shewanella oneidensis (strain MR-1)* |  |  |
| 518233316 | 64.48 | 2.35 | 1 | 1 | 60.47 |  | *Chelatococcus sp. GW1* |  |  |
| Q2SEP6 | 64.48 | 3.41 | 0 | 2 | 60.82 |  | *Hahella chejuensis (strain KCTC 2396)* |  |  |
| Q4L750 | 159.73 | 8.58 | 3 | 3 | 39.94 | Alanine dehydrogenase | *Staphylococcus haemolyticus (strain JCSC1435)* | AlaDH/PNT family | Cell wall synthesis,  L-alanine catabolic process |
| S3I9T9 | 140.23 | 8.49 | 0 | 3 | 40.05 |  | *Bacillus cereus BAG2O-2* |  |  |
| G9QI85 | 80.01 | 8.92 | 0 | 4 | 39.53 |  | *Bacillus smithii 7_3_47FAA* |  |  |
| G2RW35 | 138.41 | 25.81 | 0 | 5 | 37.54 | Ketol-acid reductoisomerase | *Bacillus megaterium WSH-002* | Ketol-acid reductoisomerase family | Isoleucine and valine biosynthetic process |
| G2RS45 | 62.25 | 24.04 | 0 | 3 | 33.40 | Cysteine synthase | *Bacillus megaterium WSH-002* | Cysteine synthase/cystathionine beta-synthase family | Cysteine biosynthetic process from serine |
| A0A072NT02 | 57.20 | 6.4 | 0 | 2 | 35.93 | Diaminopimelate dehydrogenase | *Bacillus azotoformans MEV2011* | Diaminopimelate dehydrogenase family | Lysine biosynthetic process via diaminopimelate |
| I8AFC3 | 71.49 | 7.5 | 1 | 2 | 55.85 | 5-carboxymethyl-2-hydroxymuconate semialdehyde dehydrogenase | *Bacillus macauensis ZFHKF-1* | Aldehyde dehydrogenase family | 4-hydroxyphenylacetate catabolic process |
| W3AAS3 | 125.65 | 6.42 | 1 | 3 | 56.35 | 1-pyrroline-5-carboxylate dehydrogenase | *Planomicrobium glaciei CHR43* |  | Glutamate biosynthetic process, proline catabolic process to glutamate |
| B7GFV3 | 60.74 | 6.21 | 0 | 4 | 56.70 |  | *Anoxybacillus flavithermus (strain DSM 21510 / WK1)* |  |  |
| A0A0C1YCM3 | 42.70 | 11.38 | 0 | 7 | 58.60 |  | *Bacillaceae bacterium MTCC 8252* |  |  |
| A0A098ENF2 | 42.70 | 7 | 0 | 4 | 56.42 |  | *Planomicrobium sp. ES2* |  |  |
| A0A098EN12 | 66.02 | 9.35 | 0 | 2 | 33.86 | Acryloyl-CoA reductase electron transfer subunit beta | *Planomicrobium sp. ES2* | ETF alpha-subunit/FixB family | Electron carrier activity/ FMN binding |
| Q5KVP7 | 50.61 | 9.94 | 0 | 2 | 36.65 | Ferredoxin--NADP reductase | *Geobacillus kaustophilus (strain HTA426)* | Ferredoxin--NADP reductase type 2 family | Oxidoreductase activity |
| I3E5A2 | 132.64 | 11.54 | 0 | 2 | 20.55 | Putative 2-cys peroxiredoxin | *Bacillus methanolicus PB1* | Peroxiredoxin family | Antioxidant activity, oxidoreductase activity |
| A0A0B6ALX6 | 128.60 | 34.78 | 2 | 4 | 20.35 | FMN reductase | *Bacillus megaterium NBRC 15308 = ATCC 14581* | Oxidoreductase family | Oxidoreductase, Alkanesulfonate catabolic process |
| A0A0A3I5F3 | 107.79 | 6.71 | 0 | 3 | 64.47 | Succinate dehydrogenase | *Lysinibacillus manganicus DSM 26584* | Oxidoreductase family | Oxidoreductase activity |
| D5E261 | 76.78 | 8.73 | 0 | 2 | 31.23 | Oxidoreductase, aldo/keto reductase | *Bacillus megaterium (strain ATCC 12872 / QMB1551)* | Aldo/keto reductase family |  |
| G2RLC8 | 69.19 | 7.43 | 0 | 2 | 40.12 | Glycerol dehydrogenase DhaD1 | *Bacillus megaterium WSH-002* | Oxidoreductase family |  |
| A0A0A5I6J8 | 59.77 | 7.94 | 1 | 3 | 56.01 | MFS transporter | *Pontibacillus marinus BH030004 = DSM 16465* | MFS family | Oxidoreductase activity, phenylacetate catabolic process |
| T0CRP1 | 63.81 | 7.09 | 1 | 2 | 53.29 | Betaine-aldehyde dehydrogenase | *Alicyclobacillus acidoterrestris ATCC 49025* | Aldehyde dehydrogenase family | Oxidoreductase activity |
| W1SJY0 | 133.32 | 9.2 | 2 | 3 | 53.19 | Aldehyde dehydrogenase (NAD) family protein | *Bacillus vireti LMG 21834* |  |  |
| W7ZQX8 | 102.46 | 21.37 | 0 | 2 | 14.62 | Thioredoxin | *Bacillus sp. JCM 19047* | Thioredoxin family | Cell redox homeostasis/ glycerol ether metabolic process/ protein disulfide oxidoreductase activity |
| G2RW04 | 72.64 | 36.54 | 0 | 4 | 11.46 |  | *Bacillus megaterium WSH-002* |  |  |
| G2RIY9 | 120.46 | 30.56 | 2 | 2 | 15.47 | Peptidyl-prolyl cis-trans isomerase | *Bacillus megaterium WSH-002* | Cyclophilin-type PPIase family | Protein folding |
| G2RJG0 | 125.80 | 18.67 | 1 | 2 | 31.41 | Succinyl-CoA ligase [ADP-forming] subunit alpha | *Bacillus megaterium WSH-002* | Succinate/malate CoA ligase alpha subunit family | ATP citrate synthase activity, succinate-CoA ligase (ADP- forming) activity |
| G9QPH7 | 99.67 | 6.68 | 0 | 2 | 39.72 | Peptidase T-like protein | *Bacillus smithii 7_3_47FAA* | Peptidase family | Metallopeptidase activity |
| G2RWI8 | 97.62 | 13.96 | 0 | 3 | 33.39 | Probable manganese-dependent inorganic pyrophosphatase | *Bacillus megaterium WSH-002* | PPase class C family | Hydrolase, Inorganic diphosphatase activity |
| N0B5Y3 | 97.62 | 9.65 | 0 | 2 | 33.96 |  | *Bacillus sp. 1NLA3E* |  |  |
| G2RVB2 | 94.39 | 21.76 | 0 | 5 | 46.20 | Dihydrolipoyllysine-residue acetyltransferase component of pyruvatedehydrogenase complex | *Bacillus megaterium WSH-002* | 2-oxoacid dehydrogenase family | Acyltranferase,Transferase activity |
| W1SMG8 | 85.54 | 5.42 | 1 | 2 | 56.73 | 4-hydroxyphenylacetate-3-hydroxylase | *Bacillus vireti LMG 21834* | Oxidoreductase family | Oxidoreductase activity |
| A0A0E2HKU2 | 97.11 | 6.01 | 0 | 2 | 56.80 | Putative flotillin-like protein | *Bacillus atrophaeus UCMB-5137* | Flotilin family | Not assigned |
| Q9ZF96 | 90.84 | 14.71 | 0 | 2 | 19.89 | PhaP | *Bacillus megaterium* | ----- |  |
| 518232839 | 38.28 | 1.54 | 1 | 1 | 54.80 | Hypothetical protein | *Chelatococcus sp. GW1* | ----- |  |
| A0A0B6ASA0 | 76.45 | 22.41 | 0 | 2 | 13.32 | UPF0342 protein BG04_2859 | *Bacillus megaterium NBRC 15308 = ATCC 14581* | UPF0342 family |  |
| Q95M18 | 51.48 | 3.48 | 0 | 2 | 92.37 | Endoplasmin | *Bos taurus (Bovine)* | Heat shock protein 90 family | Chaperone, protein folding, negative regulation of apoptotic process, response to hypoxia |
| Q9USQ6 | 37.95 | 3.04 | 0 | 2 | 101.64 | Inositol-1,4,5-trisphosphate 5-phosphatase 2 | *Schizosaccharomyces pombe (strain 972 / ATCC 24843) (Fission yeast)* | Inositol 1,4,5-trisphosphate 5-phosphatase family | Lipid metabolism, protein transport |
| Q12587 | 61.99 | 6.67 | 1 | 2 | 58.08 | Cytochrome P450 52C2 | *Candida maltosa (Yeast)* | Cytochrome P450 family | Monooxygenase activity, oxidoreductase |
