# Supplementary material for: Proteomics and Metabolomics Analyses to Elucidate the Desulfurization Pathway of Chelatococcus sp
Source: PLoS One. 2016 Apr 21;11(4):e0153547. doi: 10.1371/journal.pone.0153547 (PMC4839641; doi:10.1371/journal.pone.0153547)
Supplement: S2 Table — The peptides identified by MS/MS sequencing in this proteome using PEAKS 7.0 software are shown in bold and putative conserved domains have been underlined. (DOC) [file pone.0153547.s002.doc]

**Supplementary Table S2.** The alignments of MS-MS derived peptide sequences of *Chelatococcus sp*. with the BDS enzymes reported in the databases. The peptides identified by MS/MS sequencing in this proteome using PEAKS 7.0 software are shown in bold and putative conserved domains have been underlined.

**Accession No.:** BAC41359

**Matching Protein:** DBT monooxygenase DszC

**Bacterial Species:** *Mycobacterium sp. G3*

**Putative conserved domain:** ACAD Superfamily

**MTLTDDATTAQNSR**HGDPIEVARELTR**KWQTTVVERDK**AGGSATEEREDLR**TSGLLSVTVPRHLGGWGADWPTALEVVR**EIAK**VDGSLGHLFGYH**LSTPAVIDLWGSPEQKERLLRQLAENNWWTGNASSENNSHILDWKVTATPADDGGYFFNGIKHFSSGAKGSDLLLVFGVIPEGFPQQGAIVAAAIPTTREGVQPND**DWQALGMRR**TDSGTTEFHNVAVRPDEVLGKPNAILEAFLASGRGSLFGPIVQLVFSSVYLGIARGALETAREYTRTQARPWTPAGVTQAVEDPYTIRSYGEFGIQLQAADAAAR**EAAQLLQAAWDR**GDALTS**QERGELMVQISGVKAIATQAALDVTSR**IFEVIGARGTHPKYGFDRFWRNIRTHTLHDPVSYKIAEVG**NYVLNQR**YPIPGFTS

**Accession No.:** ABE26646

**Matching Protein:** DszC

**Bacterial Species:** *Rhodococcus sp. DS-3*

**Putative conserved domain:** ACAD Superfamily

MTLSPEKEHVRPRDAADNDPVAVARGLAEKWRATAVERDRAGGSATAEREDLRASALLSLLVPREYGGWGADWPTAIEVVREIAAADGSLGHLFGYHLTNAPMIELIGSQEQEEHLYTQIAQNNWWTGNASSENNSHELDVKVSATPT**EDGGYVLNGTK**HFCSGAKGSDLLFVFGVVQDDSPQQGAIIAAAIPTSRAGVTPNDDWAAIGMRQTDSGSTDFHNVKVEPDEVLGAPNAFVLAFIQSERGSLFRPIAQLIFANVYLGIAHGALDAAREYTRTQARPWTPAGIQQATEDPYTI**RSYGEFTIALQGADAAAR**EAAHLVQTVWDKGDALTPEDR**GELMAKVSGVK**SLATNAAL**NISSGVFEVIGAR**GTHPRYGFDRFWRNVR**THSLHDPVSYK**IADVGKHTLNGQYPIPGFTS

**Accession No.:** AAT78718

**Matching Protein:** DBT monooxygenase

**Bacterial Species:** *Gordonia alkanivorans*

MTLSVEKQHVRPGDADNDPVAVARGLAEKWRATAVERDRAGGSATVEREDLR**ASGLLSLLIPR**QYGGWGADWPTAIEVVREIAAADGSLGHLLGYHLSSAPMIELFGSQEQEQRLYRQIAQNDWWTGNASSENNSHVLDWKVSASPTEDGGYLLNGTKHFCSGAKGSDLLLVFGVIQDDSPQQGAIIAAVIPTSRHGVQVNDDWAAIGMRQTDSGSTDFHSVKVEPDEVLGEPNAFIVAFIQSERGSLFAPIVQLIFANVYLGIAHGALDAAREYTRTQARPWTPAGVQQATEDPYVLRAYGEFTIALQGADAAAREAAHLLQTVWDKGDALTPEDR**GELMVK**ISGVKALATNAALDVNSGIFEVIGARGTHPKYGFDRFWRNVRTHTLHDPVSYKIADVGKHTLNGQYPIPGFTS

**Accession No.:** BAC41358

**Matching Protein:** DBTO2 HBPS desulfinase DszB

**Bacterial Species:** *Mycobacterium sp. G3*

MTTTGIDRDILAYSNCPVPNALLTALESNLLAGNGISLNVLSGAQAGLHFTYDHPAYTRFGGEIPPLISEGLRAPGRTRLLGITPLAGRQGIYVR**ADSPVTSPEQLR**GRR**VGVSGAAIR**ILTGELGDYRQLDPWRQTLIALGTWEARGLLQTLHIGGIGISDVELVRIESPGVDVPEERLEAAASVKGADLFPDVAAHQSDILSSGNVDALFTWLPWAAELEDLSGAR**VLADLGDDK**RNRYASVWTVSAQLVDERPDQVQR**LVDAAVQAGR**WAQAHPEDTVGIHAANLGVAPSAIGRGFGADFAQHLIPTLDDSALAVVDQTQQFLIDHNLLDRPVDLTQWAAPQFLTQSATGEQQ

**Accession No.:** AAP80183

**Matching Protein: DszB (plasmid)**

**Bacterial Species:** *Rhodococcus erythropolis*

**Putative conserved domain:** Periplasmic binding protein type-2 superfamily

MTSRVDPANPGSELDSAIRDTLTYSNCPVPNALLTASESGFLDAAGIELDVLSGQQGTVHFTYDQPAYTRFGGEIPPLLSEGLRAPGRTRLLGITPLLGRQGFFVRDDSP**ITAAADLAGRR**IGVSASAIRILRGQLGDYLELDPWRQ**TLVALGSWEAR**ALLHTLEHGELGVDDVELVPISSPGVDVPAEQLEESATVKGADLFPDVARGQAAVLASGDVDALYSWLPWAGELQATGARPVVDLGLDER**NAYASVWTVSSGLVRQRPGLVQR**LVDA**AVDAGLWAR**DHSDAVTSLHAANLGVSTGAVGQGFGADFQQRLVPR**LDHDALALLER**TQQFLLTNNLLQEPVALDQWAAPEFLNNSLNRHR

**Accession No.:** ABE02291

**Matching Protein:** mutant HPBS desulfinase (dszB)

**Bacterial Species:** *Gordonia sp. WQ-01A*

MAGRLSPGNPGSELDTGILDTLTYSNCPIPNALLTAWESGFLDAAGIELDILSGKQGTVHFTYDQPAYTRYGGEIPPLPSEGLRAPGRTRLLGITPILGRQGFFVGDRSPITVAADLAGRRIGVSASAIRILRGELGDYLQLDPWRQ**TLVALGSWEAR**ALLHTLEHGELDVDNVEL**VPSNSLGVDVPAEQLET**PETLKGADLFPDVAAGQAAVLDRGEVDALFSWLAWAAELEGTGARPVVDLGLDERNAYASVWTVSSELVVDRPDLVQRLVDAVVDAGLWARDHGDAVTRLHAANLGVSPDAVGHGFGVDFQQRLVPRLDPDAVALLDRTQQFLLSNQLLQEPVALDQWAAPEFLNTSLNRHR

**Accession No.:** AAU14819

**Matching Protein:** DszC

**Bacterial Species:** *Gordonia alkanivorans*

**Putative conserved domain:** ACAD Superfamily

MTLSPEK**QHVRPPDAADNDPVAVAVGLAEKVR**ATAVERDRAGGFATAEREDLRASGLLSLLVPREYGGWGADWPTAIEVVREIAAADGSLGHLFGYHLTNAPMIELIGSQEQEEHLYTQIAQNNWWTGNASSENNSHVLDWKVSATPT**EDGGYVLNGTK**HFCSGAKGSDLLFVFGVVQDDSPQQGAIIAAAIPTSRAGVTPNDDWAAIGMRQTDSGSTDFHNVKVEPDEVLGAPNAFVLAFIQSERGSLFAPIAQLIFANVYLGIAHGALDAAREYTRTQARPWTPADIQQATEDPYTI**RSYGEFTIALQGADAAAR**EAAHLLQTVWDKGDALTPEDR**GELMVK**VSGVKALATNAAL**NISSGVFEVIGAR**GTHPRYGSDRFWRNVR**THSLHDPVSYK**IADVGKHTLNGQYPIPGFTS

**Accession No.:** G2RT82

**Matching Protein:** Monooxygenase, NtaA/SnaA/SoxA/DszA family protein

**Bacterial Species:** *Bacillus megaterium WSH-002*

**Putative conserved domain:** Flavin utilizing monooxygenase superfamily

MDMKKENGNMTIKRQLKLGAIIHGVGGNMGAWRHPEILSDASVNFGFYKQQAQKAEEGKFDLVFIADGLYINEKSLPHFLNRFEPLTILSALASVTSHIGLVGTLSTSYSEPFTVARQFASLDHISSGR**AGWNVVTSPLEGSALNYGK**EHPTHDKRYRAEEFLEVTKGLWDSWEDDAFIRNKETGQFFEEKKLHRLHHKGEFFSVEGPLNIGR**SAQGQPVVFQAGSSESGK**DLAAK**TADAVFTGQDNLEEAK**AFYQDVKSRAVAQGRHENELLIFPGIGPIIGSTTEEAERKYEELSQLVTIEHALNYLGRFFDHFDFSQFPLDEAFPDLGDIGSNSFRSTTDKIKENAKKHKWTLREAALRIATPKTQFIGTPEHIANLMQQWFEEKGADGFIIHSSVPHGLDDFVEHVVPILQERGLYRTEYEGSTLRSNLQLNVPK NRYTKVKVN

**Accession No.:** H0RG75

**Matching Protein:** NADH-dependent FMN reductase DszD

**Bacterial Species: *Gordonia polyisoprenivorans NBRC 16320 = JCM 10675***

MTARPGAVESRTVPAAIDPAAFRSLMRRHAAGVSVITLESDSGPVGFTATSLASLSAEPPLVCFNIAHTSSSLTALRAAQSLVIHVLDERDRAIAER**FSRTAAER**FADASSWTTLGTGEPLLVGVQCWMRVALVGK**QDIGDHVLVVGQVTK**AGLPDGDADPVRPLIYHDGR**YHHGVPVR**EV
