# Supplementary figures and images for: Proteomics and Metabolomics Analyses to Elucidate the Desulfurization Pathway of Chelatococcus sp
Source: PLoS One. 2016 Apr 21;11(4):e0153547. doi: 10.1371/journal.pone.0153547 (PMC4839641; doi:10.1371/journal.pone.0153547)

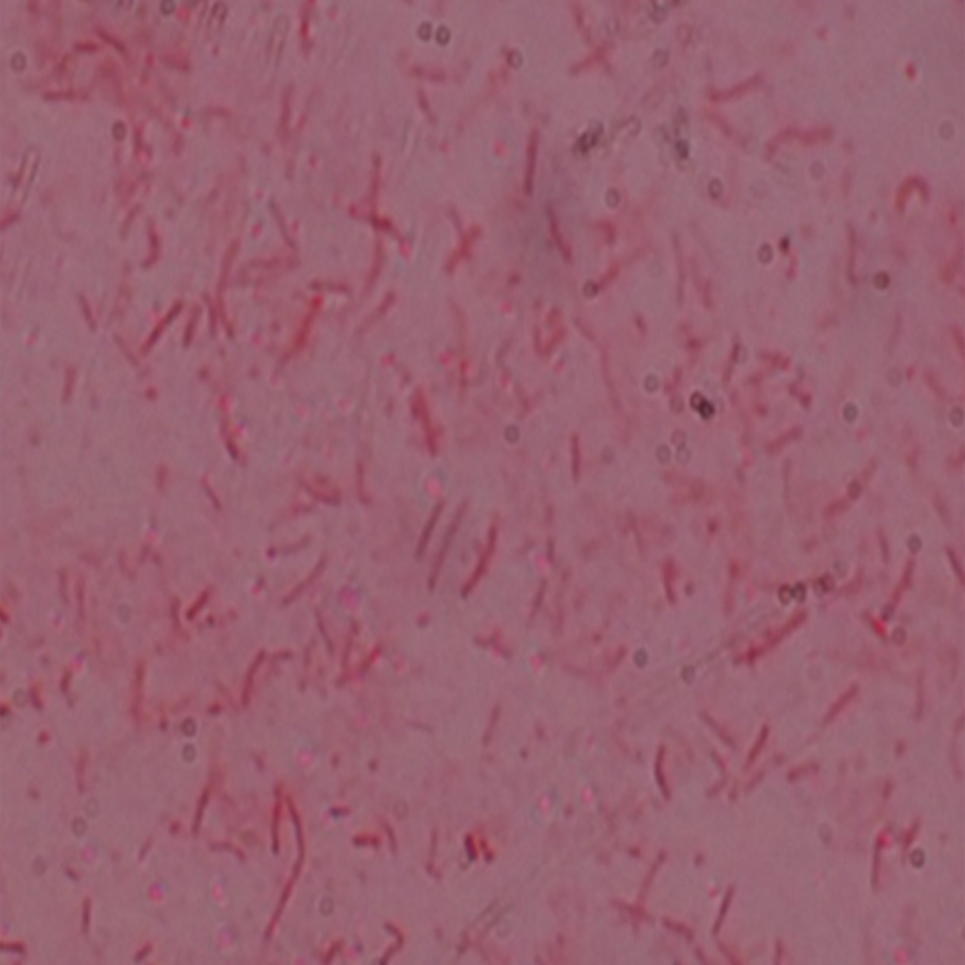

Supplement: S1 Fig — (TIF) [file pone.0153547.s003.tif]

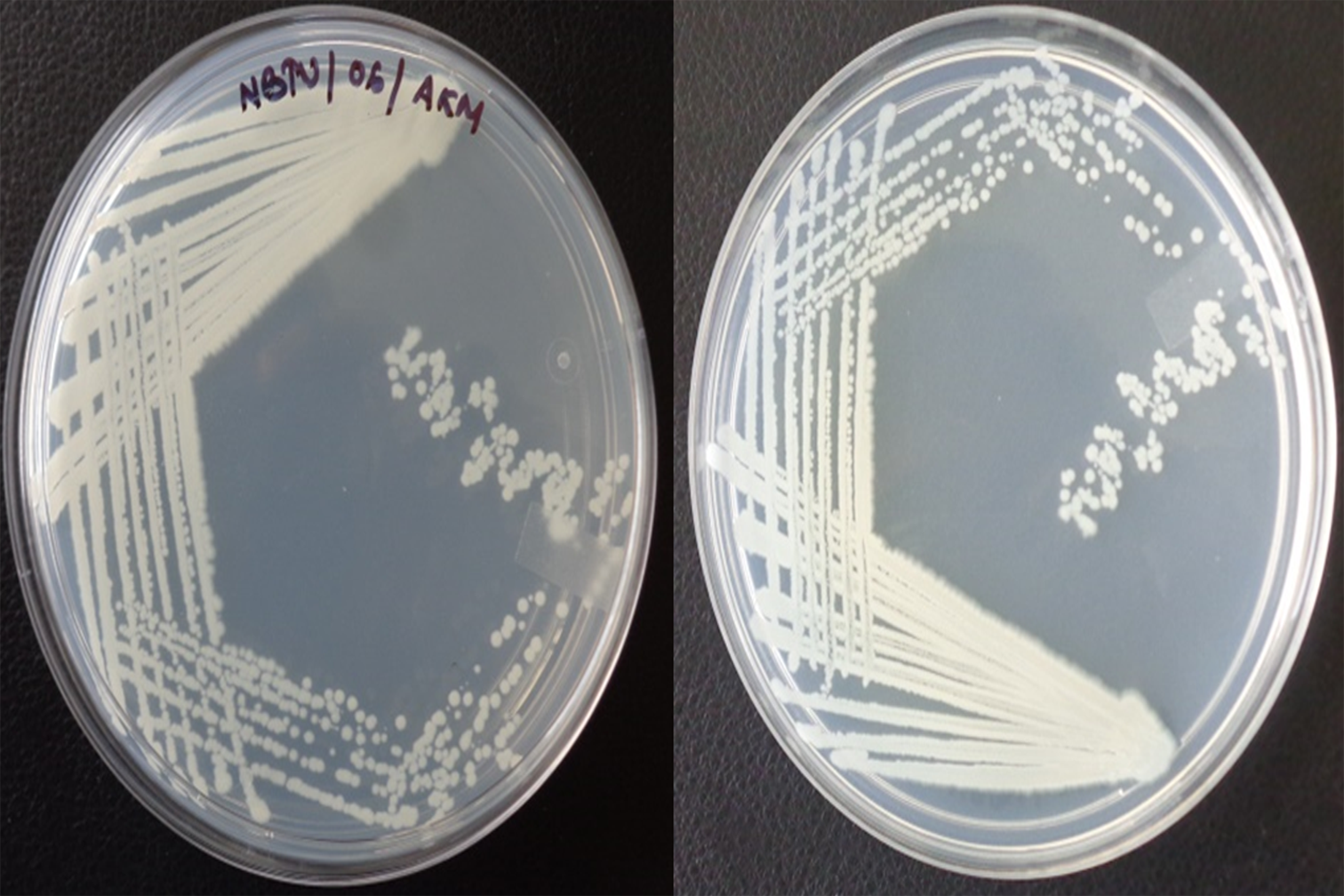

Supplement: S2 Fig — (TIF) [file pone.0153547.s004.tif]
